# Computational Exploration of Bacterial Compounds Targeting Arginine-Specific Mono-Adp-Ribosyl-Transferase 1 (Art1): A Pathway to Novel Therapeutic Anticancer Strategies

**DOI:** 10.3390/cimb47080634

**Published:** 2025-08-08

**Authors:** Nedjwa Mansouri, Ouided Benslama, Sabrina Lekmine, Hichem Tahraoui, Mohammad Shamsul Ola, Jie Zhang, Abdeltif Amrane

**Affiliations:** 1 Department of Natural and Life Sciences, Larbi Ben M’Hidi University, Oum El Bouaghi 04000, Algeria; nedjwa.mansouri@univ-oeb.dz; 2 Higher National School of Forest, Khenchela 40000, Algeria; 3 Biotechnology, Water, Environment and Health Laboratory, Abbes Laghrour University, Khenchela 40000, Algeria; 4 Laboratory of Biomaterials and Transport Phenomena (LBMTP), University Yahia Fares, Médéa 26000, Algeria; hichemm.tahraouii@gmail.com; 5 Laboratoire de Génie des Procédés Chimiques, Département de Génie des Procédés, Faculté de Technologie, Université Ferhat Abbas, Sétif-1, Sétif 19000, Algeria; 6 Univ Rennes, Ecole Nationale Supérieure de Chimie de Rennes, CNRS, ISCR—UMR6226, 35000 Rennes, France; abdeltif.amrane@univ-rennes.fr; 7 Department of Biochemistry, College of Science, King Saud University, Riyadh 11451, Saudi Arabia; mola@ksu.edu.sa; 8 School of Engineering, Merz Court, Newcastle University, Newcastle upon Tyne NE1 7RU, UK; jie.zhang@newcastle.ac.uk

**Keywords:** ART1, actinomycetes, anticancer activity, molecular docking, ADMET, molecular dynamics simulation

## Abstract

Cancer is a multifaceted and life-threatening disease characterized by the unregulated proliferation of malignant cells. Developing new therapies and diagnostic methods for cancer remains a critical focus of research. Proteins involved in cancer progression are being targeted to facilitate the discovery of effective biological treatments. Among these, the ART1 protein plays a critical role in promoting cancer progression, establishing it as a key target for drug therapy. Actinomycetes, known for their anticancer activity, were explored in this study for their potential to inhibit ART1. One hundred bioactive secondary metabolites derived from actinomycetes were subjected to in silico screening to evaluate their potential anticancer activity through inhibition of ART1. The three-dimensional structure of ART1 was generated using the SWISS-MODEL tool and validated through the Save server 6.0 and ProSa web. The structural stability of the ART1 protein was evaluated through molecular dynamics analysis using the iMod server. The potential active sites within the ART1 structure were mapped using the Computed Atlas of Surface Topography of Proteins (CASTp). Molecular docking and protein–ligand interaction studies were performed using AutoDock Vina. Additionally, pharmacophore modeling was conducted using the Pharmit server to identify promising compounds. Toxicity predictions and in silico drug-likeness assessments were carried out using Swiss-ADME and ADMET Lab which evaluate Absorption, Distribution, Metabolism, Excretion, and Toxicity (ADMET) properties. Molecular dynamics simulations results for the ART1 protein demonstrated high stability over time. Additionally, resistomycin, borrelidin, tetracycline, and oxytetracycline were identified as the top-ranking ligands, exhibiting binding energies between −8.9 kcal/mol and −9.3 kcal/mol. These ligands exhibited favorable pharmacophore profiles, drug-likeness, and ADMET properties, indicating their potential safety and efficacy in humans. In conclusion, the selected actinomycete-derived ligands show promise for further research and development as potential anticancer agents targeting ART1.

## 1. Introduction

Cancer remains one of the most pressing global health challenges and continues to be a leading cause of mortality worldwide [1,2]. Among men, the most commonly diagnosed cancers include prostate, lung, bronchial, colorectal, and bladder cancers, whereas in women, breast, lung, bronchial, colorectal, uterine, and thyroid cancers are the most prevalent [3]. Numerous enzymes play crucial roles in tumor development and progression, making them attractive targets for the development of novel therapeutic agents.

Mono-ADP-ribosyltransferases (mono-ARTs) represent a class of enzymes that catalyze the transfer of a single ADP-ribose moiety from nicotinamide adenine dinucleotide (NAD^+^) to specific target proteins, thereby modulating their function and activity. Among these, Arginine-Specific Mono-ADP-Ribosyltransferase 1 (ART1) is particularly noteworthy. ART1 is primarily expressed in epithelial and immune cells, as well as in select tissues [4]. To date, seven mono-ART enzymes (ART1 to ART7) have been identified in mammals, with five of them—ART1, ART2, ART5, ART6, and ART7—exhibiting specificity for arginine residues [4]. In humans, ART1 is typically expressed at low levels under normal physiological conditions [5]. However, its overexpression has been increasingly associated with various pathological states, including numerous forms of cancer [5,6].

ART1, a glycosylphosphatidylinositol (GPI)-anchored tumor cell surface protein, has been implicated in cancer progression [7]. It modulates immune cell function by catalyzing the mono-ADP-ribosylation of the P2X7 receptor on CD8^+^ T cells, ultimately leading to T cell apoptosis [8]. Meta-iodo-benzyl-guanidine (MIBG) has been reported as an ART inhibitor that suppresses interleukin-6 (IL-6) release through the inhibition of ART1 activity [9].

Moreover, ART1 has been associated with enhanced tumor development and metastasis in colorectal cancer (CRC) [10]. Lin et al. [9] demonstrated that silencing ART1 expression significantly reduced IL-6-induced proliferation of colorectal cancer cells. This effect was linked to decreased levels of glycoprotein 130 (gp130), a key signal transducer in the IL-6 signaling pathway, suggesting that ART1 may facilitate colorectal cancer progression by modulating inflammation-associated signaling cascades [9].

Traditional cancer treatment modalities—including chemotherapy, radiotherapy, and surgical intervention—have led to substantial advances in patient outcomes. However, challenges such as drug resistance, tumor heterogeneity, and off-target effects continue to limit their efficacy, highlighting the urgent need for more personalized and targeted therapeutic strategies [11,12]. In recent years, growing attention has been directed toward natural therapeutics as an alternative or complementary approach to reduce reliance on synthetic compounds.

Nature serves as an abundant source of pharmacologically active molecules, with approximately 60% of currently approved drugs having originated from natural products [13]. These bioactive compounds are derived from a variety of sources, including animals, plants, and microorganisms. Among microbial sources, actinomycetes—a group of Gram-positive filamentous bacteria—are particularly noteworthy for their ability to produce structurally diverse and biologically potent secondary metabolites [14]. Of the roughly 23,000 bioactive microbial secondary metabolites identified to date, actinomycetes account for over 10,000 compounds [15]. These metabolites encompass a broad range of activities, including immunosuppressive, antitumor, herbicidal, pesticidal, and cosmetic applications, as well as vitamin biosynthesis [16].

To reduce dependence on synthetic compounds, natural therapeutic strategies are gaining increasing attention. Natural products—particularly secondary metabolites produced by microorganisms such as actinomycetes—represent a rich source of bioactive molecules with promising anticancer potential. Although inherently chemical, these compounds offer remarkable structural diversity and target specificity, making them highly valuable in modern drug discovery efforts. Notably, approximately 60% of currently approved pharmaceutical agents are derived from natural sources [17].

Recent advances in bioinformatics have further accelerated the exploration of natural compounds by enabling the identification of molecular targets and supporting in silico methodologies such as virtual screening, molecular docking, and pharmacokinetic prediction in cancer research [18,19,20]. These computational techniques allow researchers to predict molecular interactions, perform high-throughput screening of potential drug candidates, and simulate cancer-related processes under various therapeutic scenarios [21]. Compared to conventional in vitro and in vivo experiments, in silico approaches offer significant advantages in terms of cost-efficiency and time-effectiveness, particularly during the early stages of drug discovery and development [22].

In this study, we aim to investigate the potential of actinomycete-derived bioactive compounds as inhibitors of ART1, a key enzyme implicated in cancer progression. To this end, we employed an integrative in silico approach combining molecular modeling, docking, and pharmacokinetic analysis. Initially, homology modeling was performed to predict the three-dimensional structure of ART1, followed by validation of its stereochemical properties to ensure structural reliability. A library of secondary metabolites derived from actinomycetes was then subjected to molecular docking simulations to identify potential inhibitors capable of interacting with the catalytic site of ART1. The top-ranking candidates were further assessed for their pharmacokinetic properties and ADMET (Absorption, Distribution, Metabolism, Excretion, and Toxicity) profiles to evaluate their drug-likeness and safety. Finally, molecular dynamics simulations were carried out to examine the structural stability and dynamic behavior of the most promising enzyme–ligand complexes over time. This study provides a comprehensive computational framework for the identification of novel natural compounds with potential anticancer activity and offers new insights into the inhibition of ART1 as a targeted therapeutic strategy.

## 2. Materials and Methods

### 2.1. Selection of Target and Template Sequences

The amino acid sequence of ADP-ribosyltransferase 1 (ART1) was retrieved from the UniProt database. Template identification for homology modeling was carried out using the BLASTp algorithm [23], querying the Protein Data Bank (PDB) [24] to identify structurally homologous proteins. The PDB file of the selected template, along with its corresponding FASTA sequence, was downloaded for further analysis. Multiple sequence alignment between the target protein and the selected template was performed using Clustal Omega, available through the European Bioinformatics Institute (EBI) sequence analysis platform (https://www.ebi.ac.uk/Tools/msa/clustalo/, accessed on 25 December 2024).

### 2.2. Protein Profile Analysis

The amino acid sequence of the target protein, ART1, was submitted to the PROSITE database (http://prosite.expasy.org/, accessed on 25 December 2024) to analyze its domains, families, and functional motifs. PROSITE provides computational tools for the identification and prediction of protein profiles, facilitating the characterization of structural and functional features of the protein [25].

### 2.3. Homogy Modeling of Target Protein

Homology modeling of the ADP-ribosyltransferase 1 (ART1) protein was carried out using the SWISS-MODEL web server (https://swissmodel.expasy.org/) [26]. The quality of the generated models was evaluated using the Qualitative Model Energy ANalysis (QMEAN) score, which guided the selection of the most reliable structural model. Visualization and structural analysis of the 3D model were performed using UCSF Chimera and PyMOL [27].

### 2.4. Validation of 3D Target Protein

The structural quality of the predicted ART1 model was evaluated using a combination of computational validation tools. PROCHECK was employed to generate a Ramachandran plot, providing insights into the stereochemical quality of the model by analyzing backbone dihedral angles (https://saves.mbi.ucla.edu/, accessed on 26 December 2024) [28]. ERRAT was used to assess the model based on non-bonded atomic interaction statistics (https://saves.mbi.ucla.edu/, accessed on 26 December 2024) [29], while Verify3D evaluated the compatibility between the 3D structure and its amino acid sequence (https://saves.mbi.ucla.edu/, accessed on 26 December 2024) [30]. Additionally, ProSA-web was utilized to calculate the Z-score, offering a quantitative measure of the overall quality and reliability of the 3D structural model (https://prosa.services.came.sbg.ac.at/prosa.php/, accessed on 26 December 2024) [31].

### 2.5. Analysis of Physicochemical Characteristics

The physicochemical properties of the target protein, including molecular weight (MW), isoelectric point (pI), extinction coefficient, number of positively and negatively charged residues (+R and −R, respectively), aliphatic index (AI), grand average of hydropathicity (GRAVY), and instability index (II), were analyzed using the ProtParam tool available on the ExPASy server (https://web.expasy.org/protparam/, accessed on 26 December 2024) [32].

### 2.6. Molecular Dynamics Simulation

Molecular dynamics (MD) simulations were performed to evaluate the stability and dynamic behavior of the ART1–ligand complex. The iMODS server (https://imods.iqf.csic.es/, accessed on 26 December 2024) was used to assess the deformability and structural rigidity of the ART1 protein through normal mode analysis (NMA) in internal coordinates [33,34]. This approach provided insights into the protein’s intrinsic flexibility and overall stability. The analysis generated a range of outputs, including deformability plots, eigenvalues, B-factors, variance profiles, covariance matrices, and atomic-scale elastic network models. Additionally, residue-level indices were calculated to characterize both the magnitude and directionality of structural fluctuations within the protein complex [34].

### 2.7. Prediction of Active Site

The active site of the target protein was identified using the Computed Atlas of Surface Topography of Proteins (CASTp) server [35], which provides detailed information on pocket geometry and surface accessibility. Visualization and analysis of the predicted binding sites were performed using PyMOL version 1.7.4.5 (Schrödinger, LLC) and the Discovery Studio Modeling Environment [36], allowing for accurate inspection of the spatial orientation and topological features of the active site.

### 2.8. Preparation of Target Protein

The target protein was prepared for molecular docking by adding hydrogen atoms and assigning partial atomic charges using the AMBER ff14SB force field [37]. This step ensured appropriate protonation states and optimized the structure for docking simulations. The finalized protein model was then saved in MOL2 format, which preserves essential information such as atomic charges and bond orders, making it suitable for subsequent docking analyses.

### 2.9. Preparation of Ligands

The chemical structures of actinomycete-derived compounds were retrieved from the PubChem database (https://pubchem.ncbi.nlm.nih.gov/) accessed on 27 December 2024 using their canonical SMILES representations. Ligand preparation was carried out in UCSF Chimera by adding hydrogen atoms and assigning Gasteiger partial charges. The finalized ligand structures were then saved in MOL2 format to preserve atomic coordinates and charge information for downstream molecular docking analysis.

### 2.10. Molecular Docking

Molecular docking analyses were performed using AutoDock Vina, following the protocol described by [38]. Both the receptor and ligands were prepared in MOL2 format prior to docking. A grid box was defined around the predicted active site using AutoDock Tools, with dimensions set to (10.2 × 37.4 × 12.9) Å and centered at coordinates (20, 20, 20) Å. Meta-iodo-benzylguanidine, a well-characterized chemical inhibitor, was used as a reference compound to benchmark binding affinities. During docking simulations, ligand flexibility was allowed, whereas the protein structure was kept rigid. The resulting protein–ligand complexes were saved in PDB format for subsequent visualization and interaction analysis.

### 2.11. Visualization of Protein–Ligand Interaction

The interactions between the receptor and ligands within the binding pocket—including hydrogen bonds, hydrophobic contacts, electrostatic interactions, and van der Waals forces—were analyzed using BIOVIA Discovery Studio Visualizer [36]. This analysis provided detailed insights into the binding modes and the nature of molecular interactions stabilizing the protein–ligand complexes.

### 2.12. Biological Activity Prediction

The potential biological activities of the studied compounds were predicted using the PASS (Prediction of Activity Spectra for Substances) online server (http://way2drug.com/passonline) accessed on 28 December 2024. This tool evaluates a wide range of pharmacological effects and estimates possible toxicities based on the structural features of each compound. The results are expressed as Pa (probability of activity) and Pi (probability of inactivity). A compound is considered to have potential pharmacological activity when its Pa value exceeds its Pi value [39].

### 2.13. Pharmacophore Study

The Pharmit web platform (http://pharmit.csb.pitt.edu/) accessed on 28 December 2024 was utilized to screen compound libraries based on pharmacophore models and molecular shape filters. Pharmacophore modeling in Pharmit integrates both ligand-based and structure-based features to account for the flexibility of residues within the catalytic site. The pharmacophore features were defined according to the chemical properties of functional groups and classified as aromatic (ARO), hydrogen bond donor (HD), hydrogen bond acceptor (HA), hydrophobic (HP), negatively charged (NI), or positively charged (PI), as identified by the Pharmit server [40].

### 2.14. Drug-Likeness and ADMET Study

The drug-likeness and physicochemical properties of the selected ligands were evaluated based on Lipinski’s Rule of Five. According to these guidelines, compounds are predicted to exhibit favorable oral bioavailability if they possess no more than five hydrogen bond donors, no more than ten hydrogen bond acceptors, a molecular weight below 500 g/mol, and a calculated partition coefficient (CLogP) of less than 5 [41].

To advance promising drug candidates toward clinical development, it is essential to assess their Absorption, Distribution, Metabolism, Excretion, and Toxicity (ADMET) profiles. These pharmacokinetic parameters are critical for predicting a compound’s behavior and safety in the human body, and help eliminate molecules with undesirable physiological or toxicological properties early in the drug development process. In this study, the in silico ADMET profiles of the most promising ART1 inhibitory compounds were predicted using two widely used online platforms: SwissADME (http://www.swissadme.ch/index.php) [42] and ADMETlab (https://admetmesh.scbdd.com/) [43] accessed on 29 December 2024.

## 3. Results and Discussion

### 3.1. Selection of Target and Template Sequences

The amino acid sequence of the target protein, Arginine-Specific Mono-ADP-Ribosyltransferase 1 (ART1), was retrieved from the UniProt database (accession number: P52961). This human protein consists of 327 amino acid residues and was obtained in FASTA format for subsequent structural and functional analyses. The complete FASTA sequence is provided in the Supporting Information (Supplementary File S1).

Template selection for homology modeling was based on maximizing sequence similarity between the target protein and available structural templates. The ART1 amino acid sequence retrieved from the UniProt database was used as input for BLASTp searches against the Protein Data Bank (PDB) to identify suitable templates. Three high-confidence templates with sequence identity above 30% were identified: PDB ID 1GXY (39.92% identity, E-value = 4 × 10^−40^), 1OG4 (39.52% identity, E-value = 4 × 10^−39^), and 1OG3 (39.52% identity, E-value = 8 × 10^−39^). According to established criteria [44], sequence identities above 30%, combined with E-values approaching zero, are considered statistically significant and suitable for reliable homology-based modeling.

**Figure 1:**
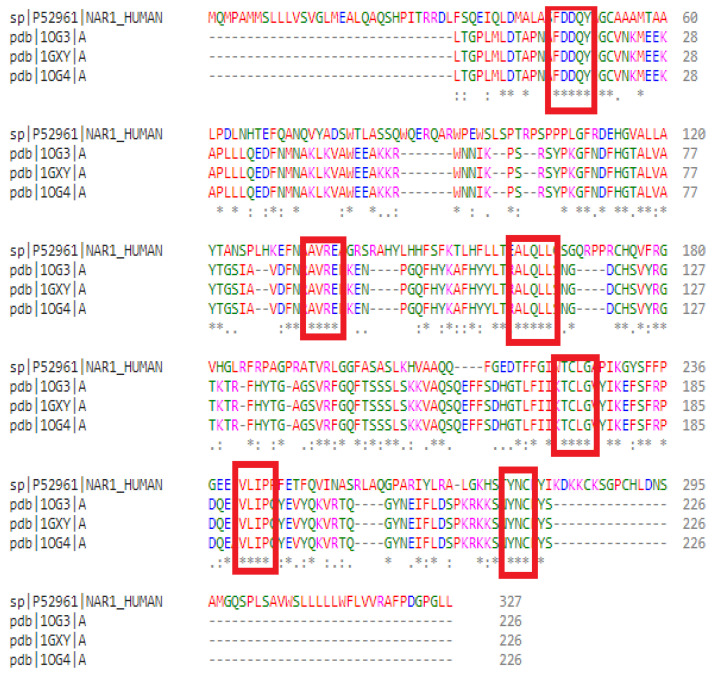
Sequence alignment performed with Clustal Omega between the query sequence and the three selected templates. Conservation levels are indicated as follows: (.) semi-conserved, (:) conserved, and (*) fully conserved. Red boxes indicate conserved amino acid regions between the target and template sequences.

The selected templates were retrieved in the FASTA format and aligned with the target sequence using Clustal Omega. Conserved amino acid regions between the target and templates were highlighted in red (Figure 1), facilitating the identification of structurally relevant regions for model construction.

### 3.2. Protein Profile Analysis

**Figure 2:**
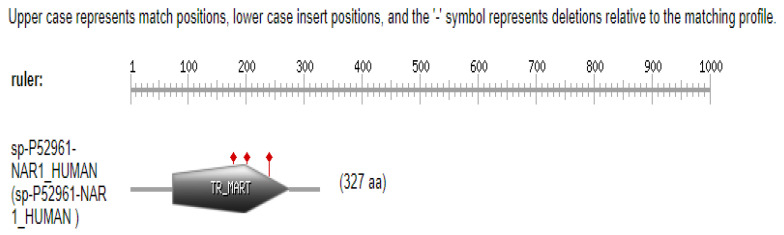
Graphical representation of Prosite result. The three red diamond-shaped markers indicate the positions of arginine (R144), serine (S202), and glutamic acid (E203), as predicted by PROSITE to constitute the protein’s active site. These residues lie within the conserved domain spanning residues 73 to 273 and are likely involved in catalytic activity or substrate binding.

The PROSITE database was employed to analyze the domain architecture, family classification, and functional sites of the ART1 protein. The analysis identified a conserved domain spanning residues 73 to 273. Within this region, three key residues—arginine (R144), serine (S202), and glutamic acid (E203)—were predicted to constitute the protein’s active site. These residues are likely involved in catalytic activity or substrate binding. A visual representation of the predicted active site is shown in Figure 2 and Supplementary File S2.

### 3.3. Homology Modeling and Validation

**Figure 3:**
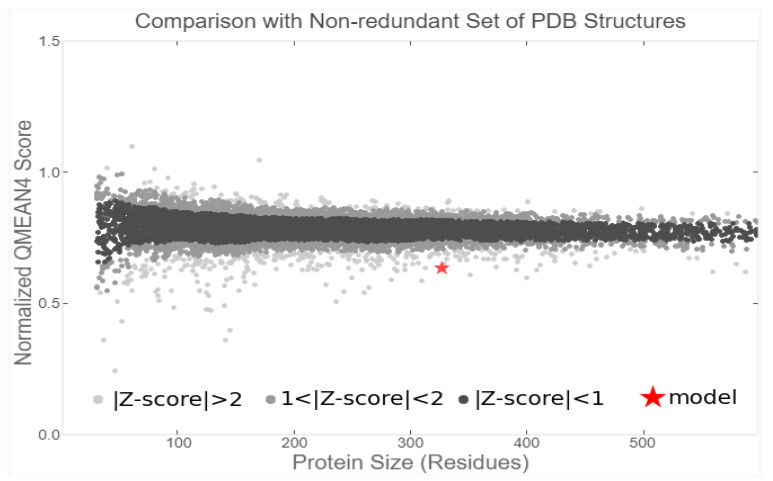
The result of QMEAN Z-score of the model.

Homology modeling of the target protein was performed using the SWISS-MODEL server, a widely used and reliable platform for comparative protein structure prediction (Figure 3 and Supplementary File S3). The structural quality of the generated model was assessed using the QMEAN Z-score, which estimates the degree of similarity between the predicted model and experimentally resolved protein structures. A QMEAN Z-score within an acceptable range supports the validity of the model, while more negative values typically reflect greater agreement with high-resolution experimental data [45].

**Figure 4:**
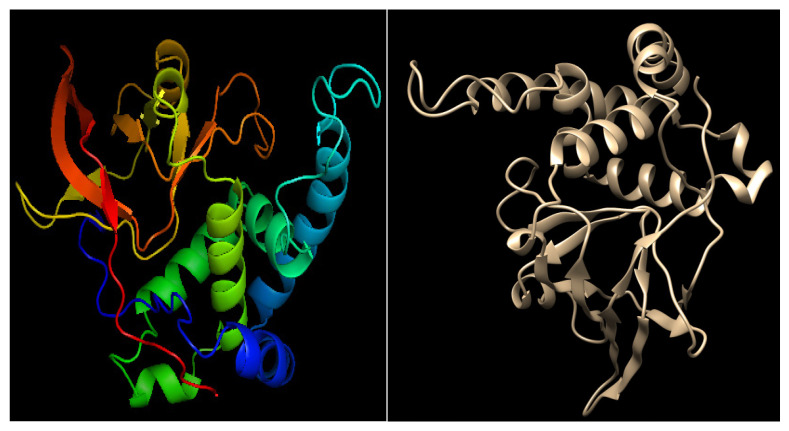
Predicted 3D structure of ART1 modeled through comparative modeling using SWISSMODEL and visualized using Discovery Studio and UCSF Chimera.

The ART1 model exhibited a QMEAN Z-score of −3.37, indicating good structural quality and reliability. The three-dimensional structure of the modeled protein was further visualized and analyzed using UCSF Chimera and PyMOL (Figure 4), allowing for detailed inspection of its structural features.

**Figure 5:**
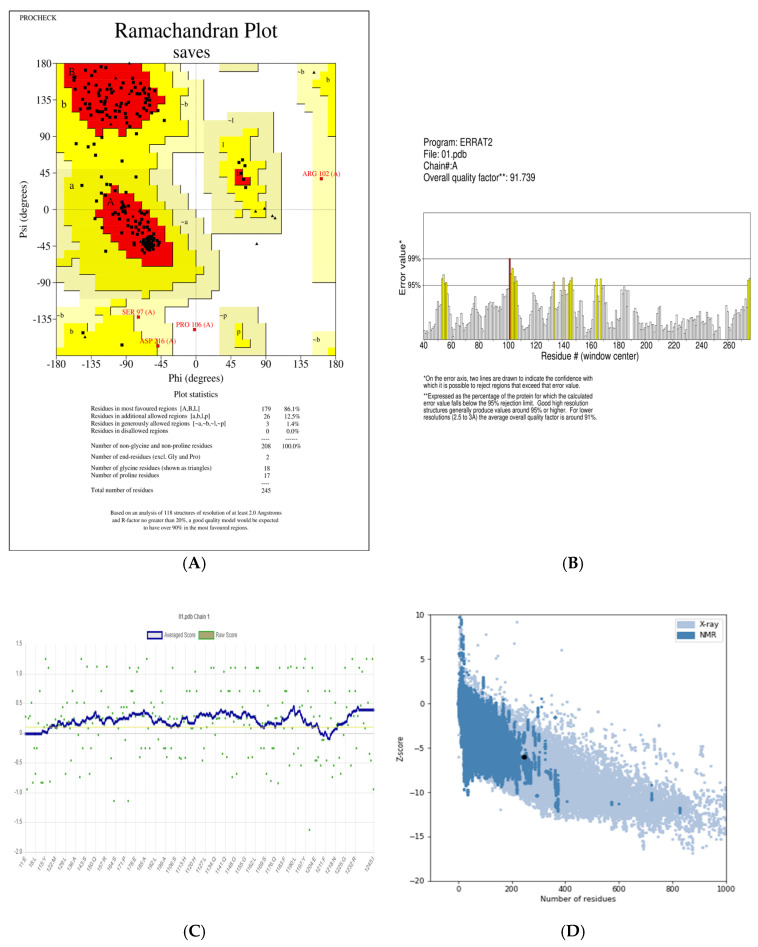
Model validation results: (**A**) **Ramachandran plot analysis** generated by PROCHECK. Black square symbols represent the φ (phi) and ψ (psi) dihedral angles of non-glycine and non-proline residues. Red regions indicate the most favored regions, yellow the additionally allowed regions, and light yellow the generously allowed regions. Residues labeled in red represent outliers. (**B**) **ERRAT evaluation**. Different colors represent the error values across the amino acid sequence; darker regions indicate higher error scores. (**C**) **VERIFY**-**3D assessment**. The yellow line shows the 3D-1D compatibility score along the protein sequence; regions above the threshold line are considered structurally acceptable. (**D**) **ProSA analysis**. The black point represents the overall Z-score of the model, indicating its quality compared to experimentally solved structures.

To assess the structural quality of the predicted ART1 model, multiple bioinformatics tools were employed, as illustrated in Figure 5 and detailed in Supplementary File S4. Stereochemical validation using a Ramachandran plot revealed that 86.1% of the amino acid residues were located in the most favored regions, 12.5% in additionally allowed regions, 1.4% in generously allowed regions, and none in disallowed regions. This analysis serves as a standard criterion for evaluating the reliability of protein structural models. The Ramachandran plot provides a visual representation of the backbone dihedral angles (ϕ–ψ) of all residues in the protein structure, excluding terminal residues. Glycine residues, due to their high conformational flexibility, are represented as triangles to indicate their ability to occupy otherwise restricted regions of the conformational space [46]. The most favorable regions of the plot correspond to well-defined secondary structural elements, typically distributed as follows: (1) β-sheet conformations in the upper-left quadrant, (2) right-handed α-helices in the lower-left quadrant, and (3) left-handed α-helices in the upper-right quadrant [47]. A model is generally considered reliable when more than 80% of residues fall within the favored regions [48]. Based on these criteria, the predicted ART1 model demonstrates high structural quality and reliability (Figure 5).

To further validate the structural integrity of the predicted model, the ERRAT tool was employed. ERRAT assesses the quality of protein structures by analyzing non-bonded atomic interactions and identifying potential error regions using a squared error function [49,50]. A score above the commonly accepted threshold of 80.00 is indicative of a high-quality and reliable model [51]. In this study, the ERRAT analysis produced a quality score of 91.7391, confirming the overall accuracy and robustness of the predicted ART1 structure (Figure 5).

To further assess the structural integrity of the predicted ART1 model, the ERRAT validation tool was employed. ERRAT evaluates the quality of protein structures by analyzing non-bonded atomic interactions and identifying regions with potential errors using a squared error function [49,50]. A commonly accepted quality threshold is 80.00, with higher scores reflecting greater structural reliability [51,52]. In the present study, the predicted model achieved an ERRAT score of 91.7391, indicating a high-quality structure and reinforcing the reliability of the homology model (Figure 5).

To further evaluate the overall structural accuracy of the predicted model, ProSA-web was employed. This tool assesses model quality by comparing the predicted structure’s potential energy to that of experimentally determined protein structures in the Protein Data Bank (PDB) [53,54]. ProSA is widely recognized for its ability to detect structural inconsistencies and evaluate the overall reliability of protein models [31]. The ART1 model yielded a Z-score of −6.4, which falls within the range typically observed for high-quality experimentally resolved structures. More negative Z-scores reflect better agreement with native protein conformations, as low-energy residue environments contribute significantly to the stability of the tertiary structure [55].

### 3.4. Analysis of Physicochemical Characteristics

The physicochemical properties of the modeled ART1 protein were analyzed using the ExPASy ProtParam server. The protein consists of 327 amino acid residues, with a calculated molecular weight of 36,334.67 Daltons. The theoretical isoelectric point (pI) was determined to be 8.53, suggesting that the protein is slightly basic under physiological conditions. The pI corresponds to the pH at which the protein carries no net electric charge, typically resulting in minimal solubility and mobility during isoelectric focusing. At this point, proteins are typically compact and structurally stable [56]. The analysis also revealed the presence of 26 negatively charged residues (Asp and Glu) and 29 positively charged residues (Arg and Lys), contributing to the protein’s overall charge distribution. The instability index was calculated at 46.08, exceeding the threshold of 40 and thus categorizing the protein as unstable in vitro [57]. The aliphatic index (AI), which reflects thermal stability based on the relative volume of aliphatic side chains (Ala, Val, Ile, and Leu), was found to be 82.17. This relatively high AI suggests that the protein may possess significant thermal stability [58]. Finally, the grand average of hydropathicity (GRAVY) was calculated to be −0.113, indicating a predominantly hydrophilic nature. Lower GRAVY values are generally associated with enhanced solubility and favorable behavior in aqueous environments.

### 3.5. Molecular Dynamic Simulation

**Figure 6:**
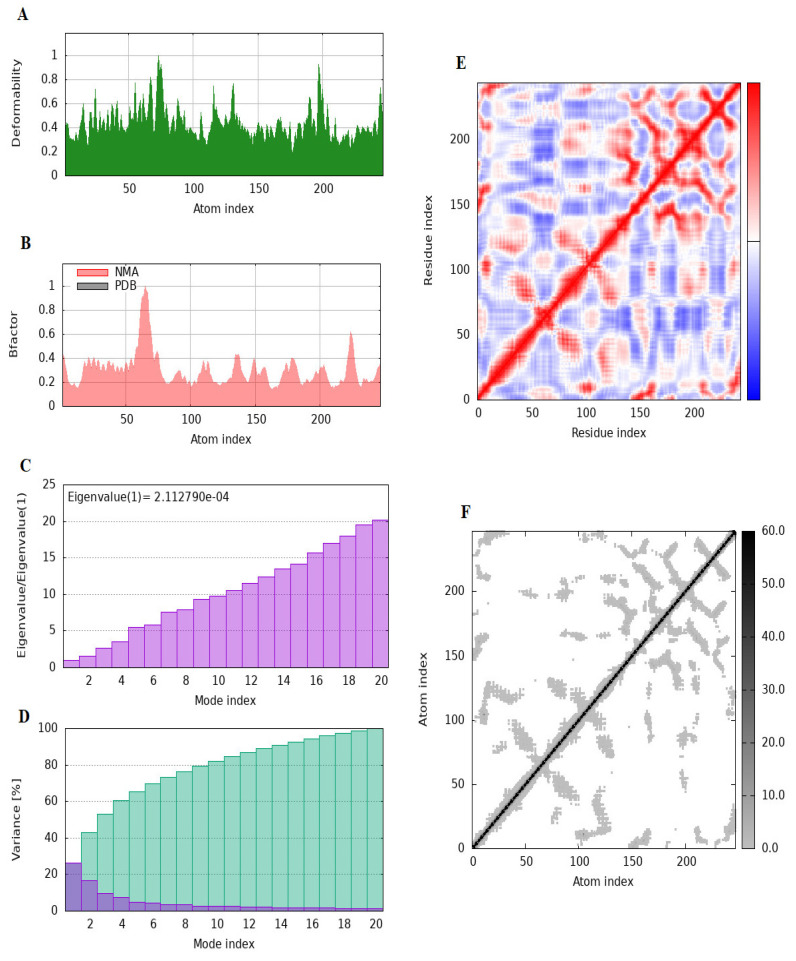
Molecular dynamics simulation results of the ART1 protein using iMODS: (**A**) deformability graphs, (**B**) B-factor plots, (**C**) eigenvalue plots, (**D**) variance maps, (**E**) covariance matrix plots, and (**F**) elastic network representation.

Molecular docking provides a static snapshot of ligand positioning within a protein’s active site, offering an initial perspective on potential binding interactions. However, it does not account for the dynamic nature of proteins and ligands under physiological conditions. To gain a more comprehensive understanding of protein–ligand interactions and their temporal stability, molecular dynamics (MD) simulations are widely employed. These simulations enable the exploration of conformational flexibility and provide insights into the stability and behavior of complexes in biologically relevant environments. Studies have shown that a high docking score does not necessarily correlate with ligand efficacy, as some compounds may dissociate from the binding site during simulation. This underscores the importance of MD simulations as a critical complement of molecular docking, providing a more realistic assessment of binding stability and molecular behavior over time [59]. In this study, the dynamic behavior of the ART1 protein was analyzed using the iMODS server (Figure 6). iMODS applies normal mode analysis (NMA) to evaluate internal molecular motions and predict conformational changes with minimal computational cost [33]. The simulation output provided detailed data on key dynamic parameters, including deformability, B-factor distribution, eigenvalues, variance, covariance matrix, and elastic network properties. These results offered valuable insights into the intrinsic flexibility and structural dynamics of ART1, a protein implicated in cancer progression.

The deformability plot (Figure 6A) presents the flexibility of individual residues based on normal mode calculations. Peaks in the green curve indicate regions with high flexibility, while valleys represent more rigid regions. The ART1 protein exhibits several highly flexible regions, particularly at residue indices around 50, 150, and 225, which likely correspond to loop regions or terminal domains that facilitate structural rearrangements. These flexible regions are essential for protein–ligand interactions and functional adaptability in dynamic cellular environments. According to [2], the highest peaks could correspond to unstructured loops, which often contribute to dynamic interactions in biological systems (e.g., active sites of enzymes or ligand binding pockets). The relatively flat regions between the peaks indicate a well-ordered structural organization, which may play a crucial role in preserving the protein’s folding and overall stability. The rigid backbone suggests a framework that provides structural integrity, resisting deformation while allowing localized movement for biological activity [60]. The B-factor, also known as the temperature factor, quantifies atomic displacement or flexibility within a molecular structure. In molecular dynamics simulations and normal mode analysis (NMA) using iMODS, the B-factor reflects atomic fluctuations within the protein, providing insights into its flexible and rigid regions [61]. The B-factor plot results (Figure 6B) compare the theoretical B-factor from NMA (red) with experimental crystallographic B-factors (gray). The overlapping trend suggests that the normal mode predictions align well with experimentally observed flexibility patterns. Notably, residues near indices 50 and 200 exhibit prominent peaks, reinforcing the idea that these regions contribute significantly to ART1’s dynamic behavior. High B-factor values indicate areas prone to conformational changes, which may play a crucial role in the protein’s biological function. This computational B-factor is often compared to experimental B-factors obtained from X-ray crystallography, providing validation for structural predictions [62]. B-factor analysis in iMOD is derived from normal mode calculations, where low-frequency modes correspond to large-scale collective motions of the protein, and high-frequency modes capture localized vibrations [2]. If a protein contains functionally important flexible regions, such as binding pockets or catalytic sites, they often exhibit higher B-factor values [63]. The eigenvalue plot (Figure 6C) provides insight into the energy required for conformational transitions. The first eigenvalue is 2.11279 × 10^−4^, indicating that ART1 has a relatively low energy barrier for large-scale motion. This suggests that the protein can undergo significant conformational shifts with minimal energy expenditure, an important feature for proteins that interact dynamically with other biomolecules. The low eigenvalue implies structural flexibility and functional adaptability, which could be critical for ligand binding or signal transduction. The eigenvalue plot shown above presents a normalized trend where eigenvalues are divided by the first eigenvalue (Eigenvalue (1)). This scaling allows for an intuitive comparison of how higher modes relate to the fundamental mode. The gradual increase in the eigenvalue ratio suggests that higher modes exhibit increased stiffness or energy levels, which is a common characteristic in modal analysis [64]. The variance distribution plot (Figure 6D) illustrates the relative contribution of each normal mode to the overall motion. The first few modes account for a substantial percentage of total variance, highlighting that global motions dominate the protein’s flexibility. The cumulative variance suggests that the first 10 modes contribute over 75% of the total motion, which means that the primary movements of ART1 can be well-characterized by these dominant modes. The purple bars represent the individual contribution to the variance of the low-contributing modes. They show that the first modes explain a significant part of the variance. In biological systems, these modes generally correspond to major conformational changes or dominant collective motions [2]. The steadily increasing green bars indicate how variance accumulates across successive modes. The curve indicates that a limited number of dominant modes account for the majority of structural variation, whereas higher-order modes contribute progressively less. In electron tomography or cryo-EM, this often means that the dataset can be efficiently analyzed using a reduced set of dominant modes, thereby improving computational efficiency [65]. The variance distribution of ART1 suggests that a few dominant modes govern its functionally relevant conformational dynamics, while higher modes contribute minimally. This idea is consistent with previous findings that biomolecular motions essential for function are captured in a small set of normal modes, highlighting the importance of variance analysis in protein structural biology. The covariance matrix (Figure 6E) represents correlated (red) and anti-correlated (blue) motions between residue pairs. The red diagonal line signifies strong correlated movements within the same structural domains, whereas off-diagonal blue and red regions indicate interactions between different parts of the protein. This suggests that specific residues move in a concerted fashion, which could be relevant for ART1’s functional dynamics. Regions with strong negative correlations (blue) suggest possible hinge-like motions, where one domain moves in the opposite direction relative to another, facilitating large-scale conformational changes. Regions of positive correlation (red) suggest cooperative motions between residues, implying structural rigidity or concerted domain movements. Conversely, negative correlations (blue) highlight anti-correlated motions, which often signify hinge points or flexible regions that facilitate large-scale conformational changes [2]. These insights are particularly useful in studying allosteric regulation, where distant residues communicate dynamically to modulate protein function [66]. The observed covariance patterns can help predict biologically relevant conformational changes, ligand-binding sites, and potential allosteric pathways. Such analyses have been widely used to study enzyme mechanisms, receptor activation, and drug design [67]. Furthermore, the integration of MD simulations with experimental data, such as X-ray crystallography or cryo-EM, enhances the reliability of structural models and provides a comprehensive understanding of protein dynamics [68]. The presence of strong off-diagonal correlations may also indicate a potential evolutionary adaptation to maintain function while allowing for necessary conformational flexibility. The Elastic Network Model (ENM) is a computationally efficient approach employed in molecular dynamics simulations to study the large-scale motions of biomolecules [2]. The iMod server utilizes ENM to generate a normal mode analysis (NMA), helping to predict collective motions within a protein structure. This method is advantageous because it reduces computational complexity while maintaining accuracy in representing functional motions [69]. The elastic network results (Figure 6F) provide insights into the connectivity between different residues. The black and gray intensity map represents the strength of interactions between atom pairs, where darker shades indicate stronger connections. The diagonal dominance signifies that the protein core is structurally stable, while the lighter regions suggest areas with higher flexibility, likely corresponding to functionally important sites. The localized weaker interactions near flexible residues (e.g., around residue indices 50, 100, and 200) further support the hypothesis that these regions contribute to ART1’s conformational adaptability. Flexible loop regions and terminal ends often exhibit significant displacement, while secondary structure elements, such as α-helices and β-sheets, generally exhibit greater rigidity [70]. These results align with known protein dynamics, where functional sites and allosteric regions are frequently associated with higher flexibility [2]. The identified pathways often correspond to biologically relevant conformational changes, such as domain opening and closing, hinge bending, or twisting motions. These results support the hypothesis that protein function is intimately linked to its intrinsic dynamics, a concept well supported by normal mode analysis studies [70]. These findings support the hypothesis that ART1 exhibits structural adaptability and is capable of undergoing significant conformational rearrangements. The analysis revealed that no atom displayed substantial distortion within the ART1 protein structure, suggesting a balanced combination of rigidity and flexibility, which may facilitate its catalytic activity. These insights contribute to a deeper understanding of ART1’s potential biological functions, particularly in protein–ligand interactions, signal transduction, and molecular recognition.

### 3.6. Prediction of Active Site

**Figure 7:**
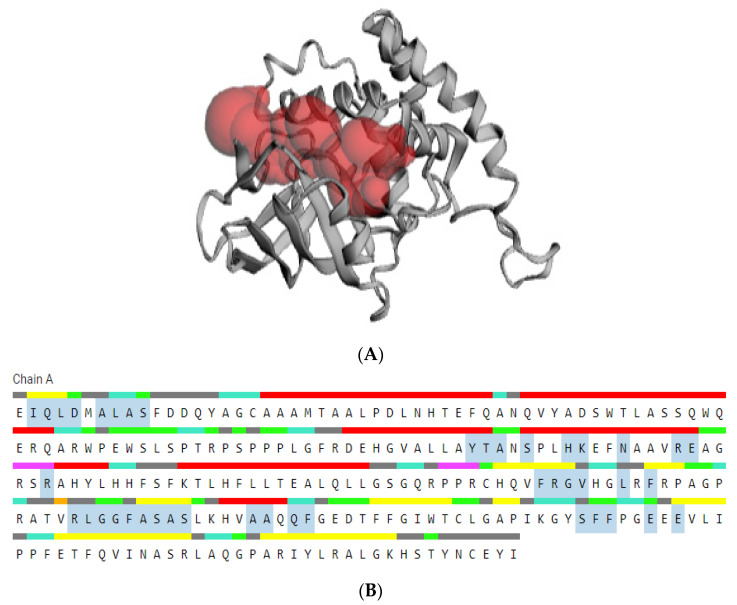
Active site of the predicted protein. (**A**) The pocket of the protein’s active site. (**B**) The marked amino acid residues in the active site of protein.

**Figure 8:**
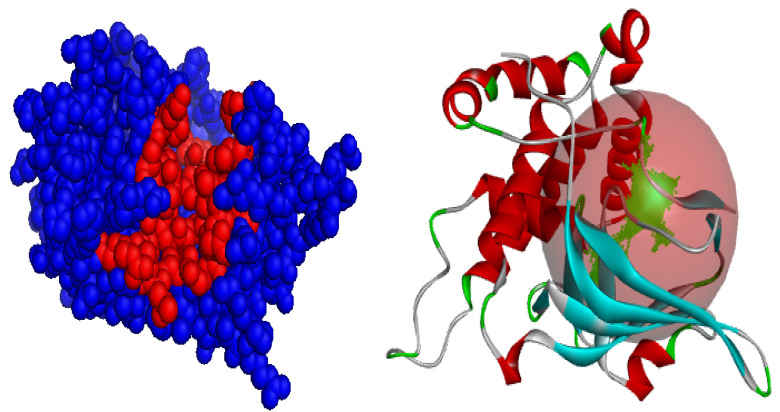
Visualization of the predicted protein’s active site using PyMOL and Discovery Studio.

The active site of an enzyme comprises specific amino acid residues responsible for substrate recognition, binding, and catalysis. Binding site residues typically establish transient interactions with the substrate, whereas catalytic residues facilitate the chemical transformation through mechanisms such as proton transfer, nucleophilic attack, or transition state stabilization [71]. In the present study, the active site of the modeled ART1 protein was identified using the CASTp server, which detects surface-accessible pockets and predicts potential ligand-binding sites based on the three-dimensional structure. The analysis revealed that 49 residues contribute to the formation of the active site (Figure 7 and Supplementary File S5). To gain further insight into the spatial organization of the active pocket, the protein structure was visualized using PyMOL and Discovery Studio Visualizer, enabling precise identification of interaction sites and cavity geometry (Figure 8 and Supplementary File S6).

Analysis of Chain A revealed a well-defined cluster of residues forming the protein’s active site, localized in a region enriched in catalytically relevant amino acids. Notably, residues such as histidine (H), serine (S), aspartate (D), lysine (K), and tyrosine (Y) were identified—amino acids commonly implicated in catalytic processes due to their ability to participate in acid–base chemistry and stabilization of transition states. Additionally, the presence of numerous hydrophobic residues, including valine (V), leucine (L), isoleucine (I), and phenylalanine (F), suggests the formation of a hydrophobic core within the binding pocket, favoring the accommodation of nonpolar or amphipathic ligands. Surrounding this core, polar and charged residues such as glutamate (E), glutamine (Q), and lysine (K) create an electrostatic environment conducive to substrate positioning and catalytic function. Collectively, these features indicate that the ART1 active site consists of a moderately sized hydrophobic cavity encased by a polar shell, which together provide both specificity and catalytic efficiency.

### 3.7. Visualization of Molecular Docking and Interaction

In this study, a molecular docking analysis was performed on 100 secondary metabolites derived from various actinomycete species to evaluate their potential inhibitory activity against the ART1 enzyme. The docking simulations were conducted using UCSF Chimera as the interface, with AutoDock Vina serving as the docking engine. To validate the docking protocol, meta-iodo-benzyl-guanidine—a well-characterized ART1 inhibitor—was used as a reference compound, yielding a binding energy of −6.1 kcal/mol. Following successful validation, the actinomycetal compounds were docked against the ART1 model. Among the tested molecules, four compounds demonstrated the most favorable binding affinities, with binding energies ranging from −8.9 kcal/mol to −9.3 kcal/mol. Resistomycin exhibited the strongest interaction with ART1, displaying a binding energy of −9.3 kcal/mol. This was followed by borrelidin and tetracycline, both with binding energies of −9.0 kcal/mol, and oxytetracycline with −8.9 kcal/mol (Table 1). These values suggest stronger and potentially more stable interactions with the ART1 active site compared to the reference molecule, highlighting these compounds as promising candidates for further investigation.

**Figure 9:**
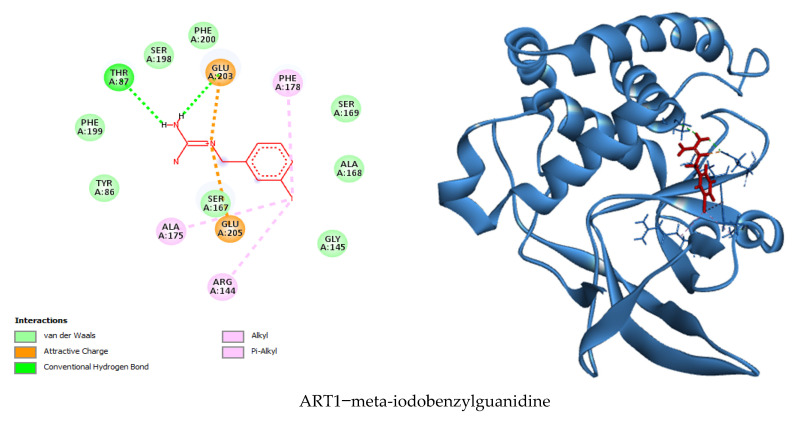

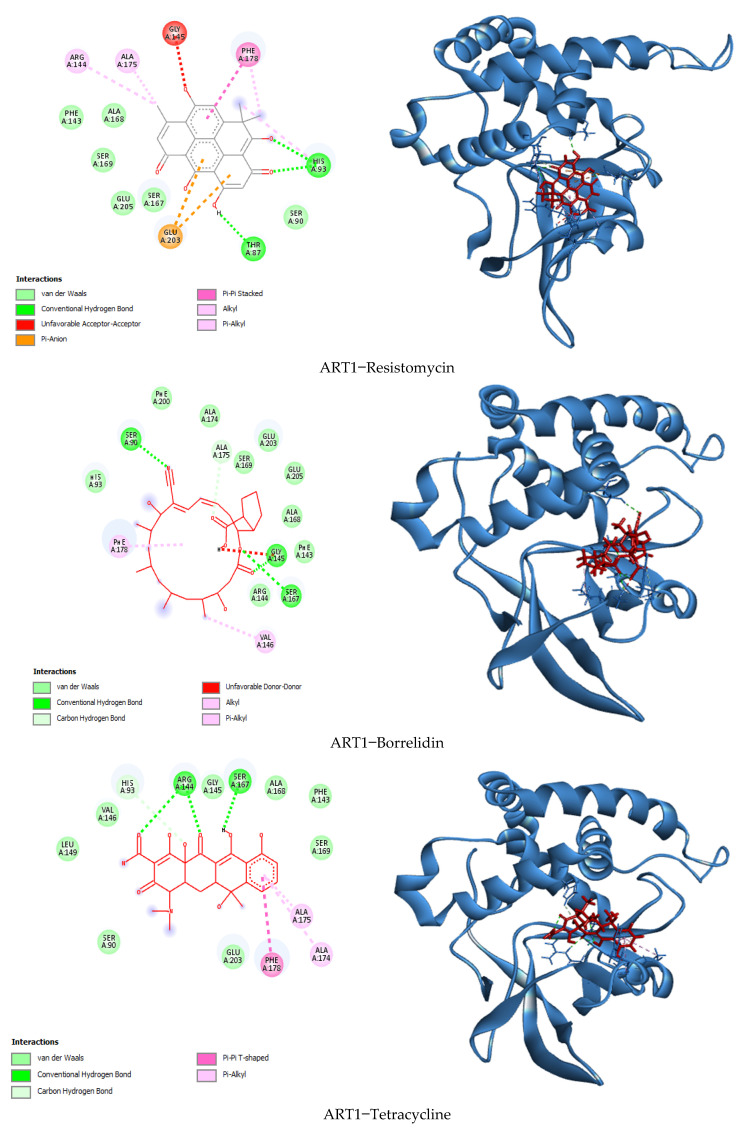

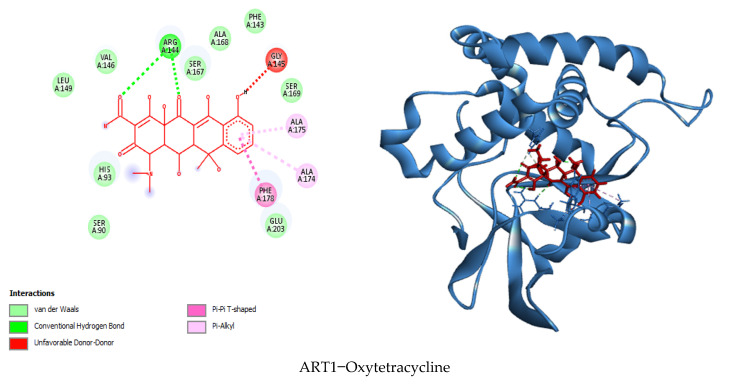
Two- and three-dimensional visualizations of the interactions between ART1 (in blue) and the top-ranked ligands (in red), emphasizing key binding features.

The molecular docking analysis determined the binding affinities of four secondary metabolites, resistomycin, borrelidin, tetracycline, and oxytetracycline, with the enzyme ART1, with binding energies between −8.9 and −9.3 kcal/mol, indicating a stronger binding affinity than the reference molecule meta-iodo-benzyl-guanidine (−6.1 kcal/mol), suggesting that these metabolites interact more effectively with the enzyme. The docking results were further examined by analyzing the types of interactions involved, including hydrogen bonding, hydrophobic interactions, electrostatic interactions, and van der Waals forces (Figure 9, Supplementary File S7), resistomycin demonstrated the strongest binding with the enzyme, forming hydrogen bonds with Thr87, His93, and His93, hydrophobic bonds with His93, Phe178, Ala175, and Arg144, and electrostatic bonds with Glu203 and Glu205. Additionally, it formed 16 van der Waals interactions, including residues like Ser90, Ser167, Glu205, Phe143, Ser169, and Ala168, leading to its highest binding energy of −9.3 kcal/mol. Borrelidin and tetracycline both showed similar binding energies of −9.0 kcal/mol, with borrelidin forming hydrogen bonds with Gly145, Ser167, Ser90, and Ala175, and hydrophobic bonds with Val146 and Phe178, while tetracycline interacted with Ser167, Arg144, His93, Ala175, and Phe178. Although borrelidin and tetracycline did not establish electrostatic bonds, they exhibited significant van der Waals interactions with residues such as Arg144, Phe143, Ala168, Glu205, Glu203, Ser169, and Ala174. Oxytetracycline, with a binding energy of −8.9 kcal/mol, exhibited fewer hydrogen bonds and van der Waals interactions, leading to a slightly lower binding affinity compared to the other three metabolites. Meta-iodo-benzyl-guanidine, serving as a reference, had only two hydrogen bonds and a relatively weaker set of interactions, which could explain its lower binding energy. Overall, the analysis suggests that the key residues interacting with the enzyme ART1 are Thr87, His93, Phe178, Ala175, Arg144, Glu203, and Ser167, which play a critical role in the binding and stability of the enzyme–ligand complexes. These findings suggest that resistomycin is the most promising candidate for further experimental validation, followed closely by borrelidin, tetracycline, and oxytetracycline, all of which could potentially serve as enzyme modulators.

### 3.8. Prediction of Biological Activity

The PASS (Prediction of Activity Spectra for Substances) online tool was employed to evaluate the anticancer potential of the selected actinomycete-derived compounds. This tool utilizes Molecular Neighborhood Analysis (MNA) descriptors to estimate two key probabilities: Pa (probability of activity) and Pi (probability of inactivity), both ranging from 0.000 to 1.000. A higher Pa relative to Pi suggests a stronger likelihood that the compound will exhibit the predicted biological activity [72]. Widely applied in preclinical drug discovery, PASS enables early-stage screening of compounds based on their chemical structures, facilitating the identification of molecules with potential therapeutic relevance.

In this study, PASS analysis was conducted on the four top-scoring ligands identified through molecular docking (Table 2). The results indicated that resistomycin and borrelidin displayed Pa values exceeding 0.7, a threshold commonly considered indicative of strong biological activity. Such values typically suggest structural similarity to known pharmacologically active compounds, reinforcing their potential as effective anticancer agents. These findings are consistent with previously reported experimental data, lending further support to their relevance in cancer drug development.

Tetracycline exhibited a Pa value in the range of 0.5–0.7, which indicates moderate confidence in its anticancer potential. While such values suggest that the compound may display biological activity, its efficacy may be limited or context-dependent, warranting further investigation.

In contrast, oxytetracycline presented a Pa value below 0.5, suggesting a low likelihood of known anticancer activity. Although this may reflect limited therapeutic potential within the currently established frameworks, it does not rule out novel mechanisms of action. Indeed, oxytetracycline could represent a candidate for exploratory studies aimed at identifying unconventional or previously uncharacterized anticancer pathways. Overall, the PASS predictions aligned well with known pharmacological profiles, supporting the prioritization of resistomycin and borrelidin for further experimental validation.

### 3.9. Pharmacophore Study

**Figure 10:**
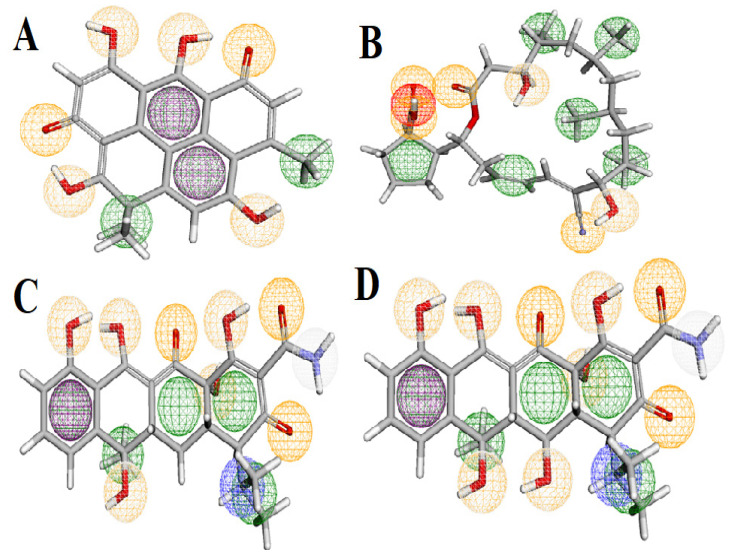
Results of pharmacophore study. Hydrophobic interactions are shown in green, negative ions in red, positive ions in blue, hydrophobic bonds in purple, and hydrogen donors and acceptors in white and orange, respectively ((**A**): resistomycin, (**B**): borrelidin, (**C**): tetracycline, (**D**): oxytetracycline).

A pharmacophore is a collection of spatial and electronic features crucial for interacting with a macromolecular target, leading to a biological response [73]. It defines the key characteristics required for molecular interactions, which include the precise spatial arrangement of features such as hydrogen bond acceptor and donor, aromatic groups, charges, and hydrophobic regions. The Pharmit server is a widely used tool for visualizing these interactions, and it assigns distinct colors to various types of molecular features. Aromatic bonds are colored purple, indicating regions involved in π-π stacking interactions, while hydrogen bond donors are represented in white, and hydrogen bond acceptors are depicted in orange, typically around hydroxyl (-OH) or amine (-NH) groups. Hydrophobic interactions are shown in green, negative ions in red, and positive ions in blue [74]. In our study, the pharmacophore analysis provided detailed insights into the molecular features of the compounds tested for their potential as ART1 inhibitors. Resistomycin exhibited two aromatic groups, four hydrogen bond donors, six hydrogen bond acceptors, and four hydrophobic interactions. This suggests that aromaticity and hydrogen bonding are crucial for its biological activity, highlighting the compound’s capability for specific molecular recognition through π-π stacking interactions and hydrogen bonding (Figure 10). Borrelidin exhibited two hydrogen bond donors, six hydrogen bond acceptors, one negative ion, and six hydrophobic interactions. These characteristics indicate that the molecule’s flexibility, potentially enhanced by its ability to engage in both hydrogen bonding and hydrophobic interactions, may improve its adaptability to various biological targets, reinforcing its potential as a versatile inhibitor (Figure 10). Tetracycline exhibited one aromatic bond, seven hydrogen bond donors, eight hydrogen bond acceptors, one positive ion, and five hydrophobic interactions. The presence of both aromatic bonds and extensive hydrogen bonding indicates that these interactions are central to its biological function. The compound’s ability to form multiple hydrogen bonds enhances its potential for effective binding to target proteins (Figure 10). Oxytetracycline, characterized by one aromatic bond, eight hydrogen bond donors, nine hydrogen bond acceptors, one positive ion, and five hydrophobic interactions, exhibited the most optimal pharmacophore model, totaling 23 interactions. This model underscores the importance of conjugated systems for achieving higher binding affinity and suggests that this compound has the strongest potential for inhibition, with a robust set of interactions that may facilitate more stable binding to the ART1 target (Figure 10).

To validate the pharmacophore model, the reference compound meta-iodobenzylguanidine (MIBG)—a known ART1 substrate—was used as a control. The model successfully recognized and matched the key interaction features present in MIBG, including its hydrogen bond donors, positive ionic regions, and aromatic moieties. The structural alignment and pharmacophoric overlap between MIBG and the predicted active compounds confirmed that the model accurately captures the essential features required for ART1 binding. This validation step supports the reliability and predictive power of the constructed pharmacophore model for virtual screening applications.

Pharmacophore models are an essential tool for large-scale compound screening, as they apply various filters to identify promising candidates based on their spatial and physicochemical characteristics [75].

A compound that satisfies all the necessary criteria for biological activity is considered a “good” candidate. By matching compounds to a well-defined pharmacophore, it becomes possible to narrow down those most likely to exhibit the desired biological effect. These candidates can then undergo more focused pharmacokinetic studies to assess their stability and suitability for further development, including parameters such as the most favorable binding energy. This approach allows for the identification of compounds that may achieve similar biological effects at lower concentrations, improving the efficiency of drug development [76].

### 3.10. Drug-Likeness and ADMET Study

The most selective ligands inhibiting ART1 were evaluated based on Lipinski’s, Veber’s, Egan’s, and Ghose’s rules, along with their ADMET properties. The results are presented in Table 3.

Lipinski’s Rule of Five is a widely adopted framework in drug discovery for evaluating the drug-likeness and oral bioavailability potential of small molecules [77]. According to this rule, compounds are considered likely to exhibit favorable oral bioavailability if they possess a log *p* ≤ 5, a molecular weight ≤ 500 Da, no more than 10 hydrogen bond acceptors, and no more than 5 hydrogen bond donors [78]. In the present study, resistomycin, borrelidin, and tetracycline complied fully with Lipinski’s criteria, while oxytetracycline exceeded the hydrogen bond donor threshold (7 donors), indicating potential bioavailability limitations.

Topological Polar Surface Area (TPSA), a key determinant of molecular polarity and absorption, should ideally fall within the range of 20–130 Å^2^ [79]. Resistomycin (115.06 Å^2^) and borrelidin (127.85 Å^2^) met this requirement, whereas tetracycline and oxytetracycline exceeded the upper limit, suggesting potential issues with membrane permeability. All compounds exhibited a number of rotatable bonds (nRB) below 10, reflecting favorable conformational flexibility.

According to Ghose’s filter, an optimal drug candidate should have a log *p* between −0.4 and 5.6, molecular weight between 160 and 480 Da, molar refractivity between 40 and 130, and an atomic count between 20 and 70 [80]. Only resistomycin satisfied all these criteria, while the other three compounds showed deviations, indicating potential challenges in terms of drug-likeness.

Veber’s rule, which considers oral bioavailability based on ≤10 rotatable bonds and TPSA ≤ 140 Å^2^, was fulfilled by resistomycin and borrelidin, again suggesting more favorable pharmacokinetics for these molecules [81]. Egan’s rule, which evaluates bioavailability potential based on a log *p* between −1.0 and 5.8 and TPSA ≤ 130 Å^2^, also confirmed compliance for resistomycin and borrelidin [82].

Finally, Muegge’s filter, which assesses drug-likeness based on structural features and classifies compounds with a drug score between 2 and 7 as drug-like [83], identified resistomycin as a drug-like molecule, while borrelidin, tetracycline, and oxytetracycline were categorized as non-drugs.

Taken together, these analyses highlight resistomycin as the most promising candidate with favorable physicochemical and pharmacokinetic properties, fully aligning with multiple drug-likeness filters, while borrelidin shows good potential but with minor deviations. In contrast, tetracycline and oxytetracycline displayed less favorable profiles, suggesting that their pharmacological development may require structural optimization or alternative delivery strategies.

The observed discrepancies in drug-likeness parameters underscore the need for a more comprehensive evaluation of the pharmacokinetic and safety profiles of the selected compounds. Key physicochemical properties such as molecular weight and topological polar surface area (TPSA) play critical roles in determining a molecule’s ability to permeate biological membranes. Higher molecular weight and elevated TPSA are generally associated with reduced membrane permeability, whereas lower values favor improved absorption and bioavailability [84]. Similarly, the partition coefficient (LogP) reflects molecular lipophilicity, with moderate values typically indicating better passive diffusion. The balance between hydrogen bond donors and acceptors is also essential for membrane permeability, as excessive polarity can hinder transcellular transport. Furthermore, the number of rotatable bonds (nRB) is indicative of molecular flexibility; an optimal nRB supports favorable bioavailability by maintaining a balance between conformational adaptability and rigidity [84].

To address these aspects systematically, in silico prediction of ADMET (Absorption, Distribution, Metabolism, Excretion, and Toxicity) properties has become a cornerstone of early-phase drug discovery. Conducting experimental ADMET evaluations for large compound libraries is both time-consuming and resource-intensive, making computational screening tools an indispensable alternative [85]. Platforms such as SwissADME (http://www.swissadme.ch/index.php) and ADMETlab (https://admetmesh.scbdd.com/) provide rapid, reliable predictions of a compound’s pharmacokinetic behavior and potential toxicity, offering valuable guidance during the lead optimization process.

In this study, the ADMET properties of the top four candidate ligands were evaluated using these platforms. The predictive results, summarized in Table 4, offer insight into key parameters including solubility, gastrointestinal absorption, blood–brain barrier permeability, metabolic stability, hepatotoxicity, and interaction with cytochrome P450 enzymes and drug transporters. These in silico analyses facilitate the early identification of promising compounds while deprioritizing those with suboptimal pharmacokinetic or safety profiles, thus streamlining the drug development process and increasing the likelihood of downstream success.

Caco-2 permeability, assessed using human colon-derived adenocarcinoma cells, serves as a well-established in vitro model for predicting intestinal absorption due to the morphological and functional similarity of Caco-2 cells to human enterocytes [86]. Compounds with predicted permeability values above −5.15 log cm/s are typically considered to have good absorption profiles [87,88]. In this study, all tested compounds, except oxytetracycline, demonstrated Caco-2 permeability consistent with favorable intestinal absorption. Human Intestinal Absorption (HIA), an integrated parameter derived from excretion ratios in urine, bile, and feces [89], confirmed that all compounds except borrelidin achieved HIA values > 30%, supporting their classification as highly absorbable [90].

Regarding distribution, all four compounds were predicted as BBB−, indicating their inability to cross the blood–brain barrier, a characteristic commonly observed for non-lipophilic or high-molecular-weight compounds [91]. In terms of plasma protein binding (PPB), all compounds except borrelidin exhibited positive binding, suggesting efficient systemic distribution through reversible interactions with plasma proteins such as albumin and glycoproteins, which influence drug half-life and bioavailability [92,93].

Metabolic analysis revealed that tetracycline and oxytetracycline were not predicted to inhibit any cytochrome P450 (CYP450) isoforms, whereas resistomycin and borrelidin showed potential inhibition of one or more isoforms, which may influence drug–drug interactions and hepatic clearance [94,95,96]. Excretion parameters were assessed via predicted total clearance (Cl), where resistomycin, tetracycline, and oxytetracycline displayed low clearance (<5 mL/min/kg), suggesting prolonged systemic retention, while borrelidin exhibited high clearance (>15 mL/min/kg), indicating faster elimination [97].

Toxicological profiling was conducted to evaluate the compounds’ safety risks. The Ames mutagenicity test predicted non-mutagenic outcomes for all compounds except resistomycin, suggesting a favorable genetic safety profile for most candidates [98]. In carcinogenicity assessments, all four molecules tested negative, indicating no predicted risk of cancer-related toxicity [99]. However, borrelidin was flagged as a potential hERG channel blocker, which may pose cardiotoxicity risks due to the inhibition of potassium ion channels critical for cardiac repolarization [100]. The remaining compounds tested negative for hERG inhibition, indicating minimal cardiotoxic potential. In terms of hepatotoxicity, oxytetracycline was the only compound predicted to be non-hepatotoxic, while resistomycin, tetracycline, and borrelidin exhibited potential hepatotoxicity risks, underscoring the need for further in vitro and in vivo validation [101,102].

Collectively, these in silico ADMET predictions provide a comprehensive overview of the pharmacokinetic behavior and toxicity risks associated with each compound. Resistomycin and tetracycline emerged as promising candidates with favorable absorption and distribution profiles, but their potential hepatic liabilities merit additional safety evaluations. Borrelidin, while demonstrating excellent intestinal permeability and high clearance, presents concerns related to hERG inhibition and hepatotoxicity. Oxytetracycline, despite a lower permeability profile, showed the most favorable toxicity profile among the tested compounds.

## 4. Conclusions

Based on the results obtained in this study, we demonstrate the potential of actinomycete-derived bioactive compounds as inhibitors of ART1, a critical enzyme involved in cancer progression. Through an in silico approach combining molecular modeling, docking simulations, pharmacokinetic analysis, and molecular dynamics simulations, we identified several promising compounds with the potential to target ART1 effectively. The selected ligands resistomycin, borrelidin, tetracycline, and oxytetracycline showed favorable binding affinities, drug-likeness, and ADMET profiles, suggesting their potential as safe and effective therapeutic agents. Molecular dynamics simulations by the iMod server confirmed the stability of the enzyme. The analysis of key parameters, including deformability, B-factor distribution, eigenvalues, variance, covariance matrix, and elastic network properties, further supported the stability and compactness of the ART1 protein. This study highlights the potential of these actinomycete-derived compounds as anticancer agents targeting ART1, offering a promising avenue for future research and development of novel cancer therapies. Additional experimental validation and in vivo studies are necessary to confirm the efficacy and safety of these compounds, but the in silico results present a strong foundation for their potential as new anticancer drugs.

## Figures and Tables

**Table 1:** Result of molecular docking of the best ligands with the predicted ART1 model.

	Binding Energy (Kcal/mol)	Hydrogen Bonds	Hydrophobic Bonds	Electrostatic Bonds	Van der Waals Bonds	Total Number of Bonds
Meta-iodo-benzyl-guanidine	−6.1	Thr87, Glu203	Phe178, Ala175, Arg144	Glu203, Glu205	Gly145, Ala168, Ser169, Phe200, Ser198, Phe199, Tyr86, Ser167	15
Resistomycin	−9.3	Thr87, His93, His93	His93, Phe178, Phe178, Ala175, Arg144	Glu203, Glu203	Ser90, Ser167, Glu205, Ser169, Ala168, Phe143.	16
Borrelidin	−9.0	Gly145, Ser167, Ser90, Ala175	Val146, Phe178	/	Arg144, Phe143, Ala168, Glu205, Glu203, Ser169, Ala174, Phe200, His93	15
Tetracycline	−9.0	Ser167, Arg144, Arg144, His93	Ala175, Ala175, Phe178	/	Ser169, Phe143, Ala163, Gly145, Val146, Leu149, Ser90, Glu203	15
Oxytetracycline	−8.9	Arg144, Arg144	Ala174, Ala175, Phe178	/	Ser169, Phe143, Ala168, Ser167, Val146, Leu149, His93, Ser90, Glu203	14

**Table 2:** Results of biological activity prediction.

Biological Activity	Resistomycin		Borrelidin		Tetracycline		Oxytetracycline	
	Pa	Pi	Pa	Pi	Pa	Pi	Pa	Pi
Anticancer activity	0.896	0.005	0.799	0.012	0.529	0.063	0.465	0.082

PA—Probability of Activity; Pi—Probability of Inactivity.

**Table 3:** Drug-likeness characteristics of the selected molecules.

	Molar Weight (g/mol)	LogP	LogS	HBA	HBD	TPSA (Å^2^)	AMR	nRB	Lpinski	Ghose	Veber	Egan	Muegge
Resistomycin	376.36	2.89	−4.61 MS	6	4	115.06	104.73	0	Yes	Yes	Yes	Yes	Yes
Borrelidin	489.64	3.61	−2.53 S	7	3	127.85	136.66	2	Yes	No	Yes	Yes	No
Tetracycline	444.43	−0.34	−1.82 S	9	6	181.62	110.79	2	Yes	No	No	No	No
Oxytetracycline	460.63	−1.01	−1.0 S	10	7	201.85	111.95	2	No	No	No	No	No

**Table 4:** Results of ADMET study.

		Resistomycin	Borrelidin	Tetracycline	Oxytetracycline
**Absorption**	**Caco2**	Yes	Yes	Yes	No
	**HIA**	Yes	No	Yes	Yes
**Distribution**	**BBB**	No	No	No	No
	**PPB**	Yes	No	Yes	Yes
**Metabolism**	**CYP1A2 inhibitor**	Yes	No	No	No
	**CYP2C19 inhibitor**	No	No	No	No
	**CYP2C9 inhibitor**	Yes	No	No	No
	**CYP2D6 inhibitory**	No	No	No	No
	**CYP3A4 inhibitor**	Yes	Yes	No	No
**Excretion**	**Cl**	0.493 (L)	7.91 (M)	2.238 (L)	1.704 (L)
**Toxicity**	**Ames test**	Yes	No	No	No
	**Carcinogencity**	No	No	No	No
	**hERG Blockers**	No	Yes	No	No
	**H-HT**	Yes	Yes	Yes	No

## Data Availability

The original contributions of this study are available in the article and its Supplementary Materials. For additional information, please contact the corresponding authors.

## Supplementary Materials

The following supporting information can be downloaded at: https://www.mdpi.com/article/10.3390/cimb47080634/s1.
