# Supplementary material for: Computational Exploration of Bacterial Compounds Targeting Arginine-Specific Mono-Adp-Ribosyl-Transferase 1 (Art1): A Pathway to Novel Therapeutic Anticancer Strategies
Source: Curr Issues Mol Biol. 2025 Aug 8;47(8):634. doi: 10.3390/cimb47080634 (PMC12384204; doi:10.3390/cimb47080634)
Supplement: Supplementary file 1 [file cimb-47-00634-s001.zip › cimb-3728480-supplementary.pdf]

The complete FASTA sequence of the ART1 protein used in this study is provided below.
>sp—P52961—NAR1-HUMAN GPI-linked NAD(P)(+)–arginine ADPribosyltransferase 1 OS=Homo sapiens OX=9606 GN=ART1 PE=2 SV=2

MQMPAMMSLLLVSVGLMEALQAQSHPITRRDLFSQEIQLDMALASFDDQYAGCAAAMTAA
LPDLNHTEFQANQVYADSWTLASSQWQERQARWPEWSLSPTRPSPPPLGFRDEHGVALLA
YTANSPLHKEFNAAVREAGRSRAHYLHHFSFKTLHFLLTEALQLLGSGQRPPRCHQVFRG
VHGLRFRPAGPRATVRLGGFASASLKHVAAQQFGEDTFFGIWTCLGAPIKGYSFFPGEEE
VLIPPFETFQVINASRLAQGPARIYLRALGKHSTYNCEYIKDKKCKSGPCHLDNSAMGQS
PLSAVWSLLLLLWFLVVRAFPDGPGLL

Figure S1. FASTA Sequence of the ART1 Protein.

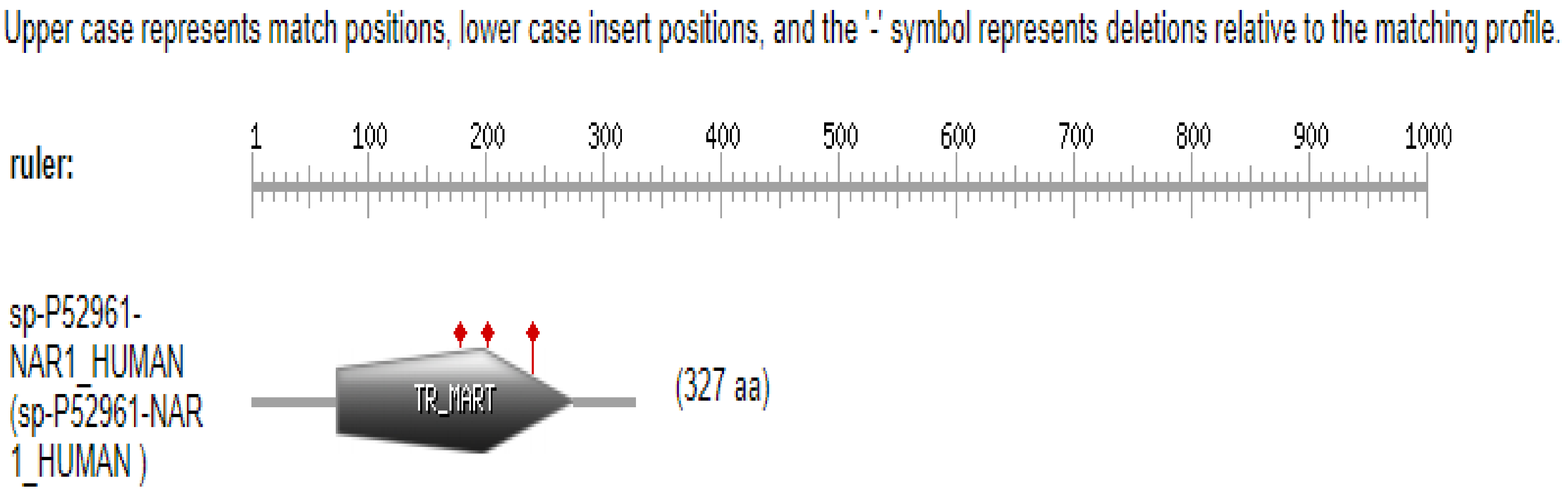

Figure S2. Graphical representation of Prosite result.

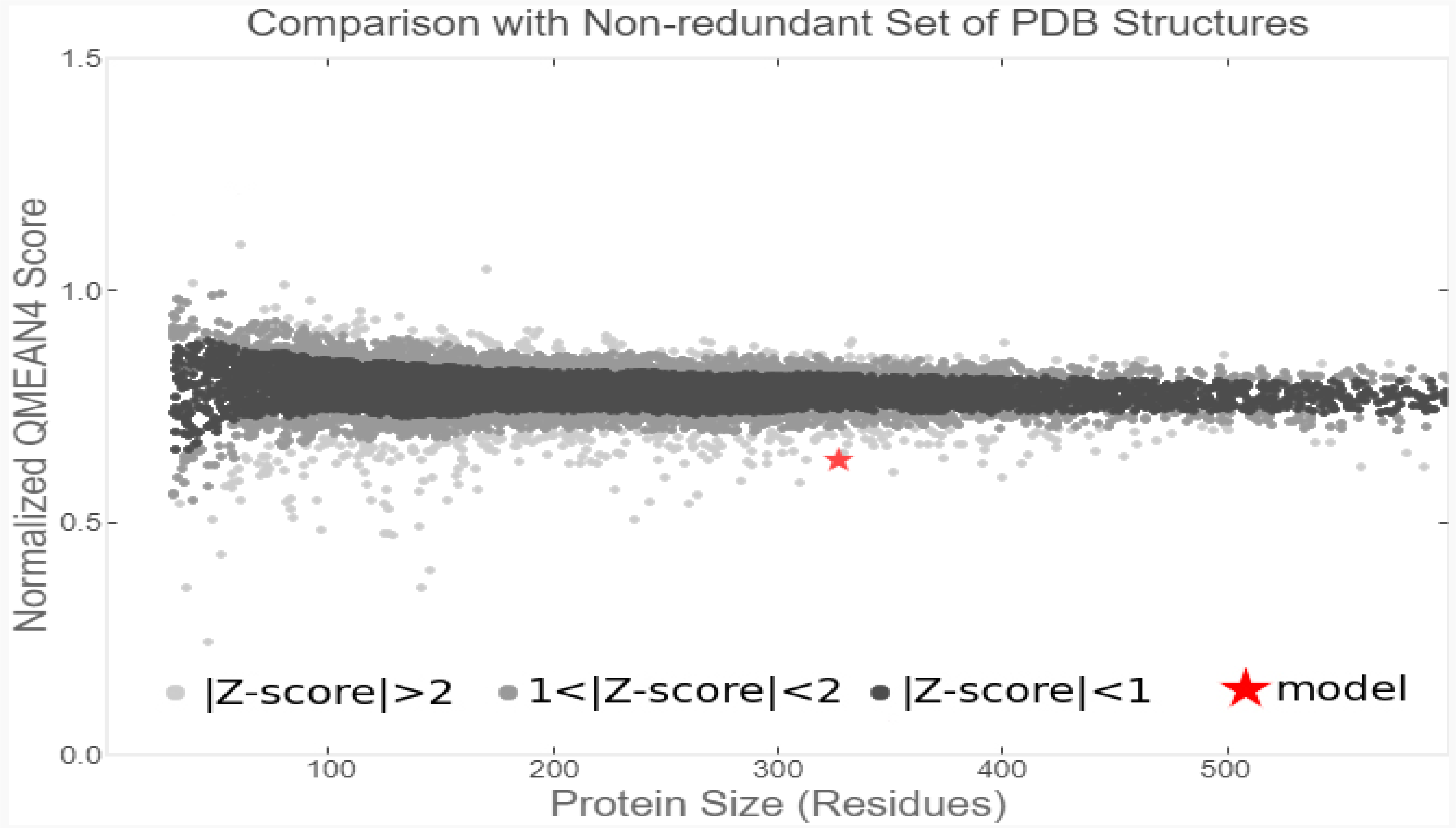

Figure S3. The result of QMEAN Z-score of the model.

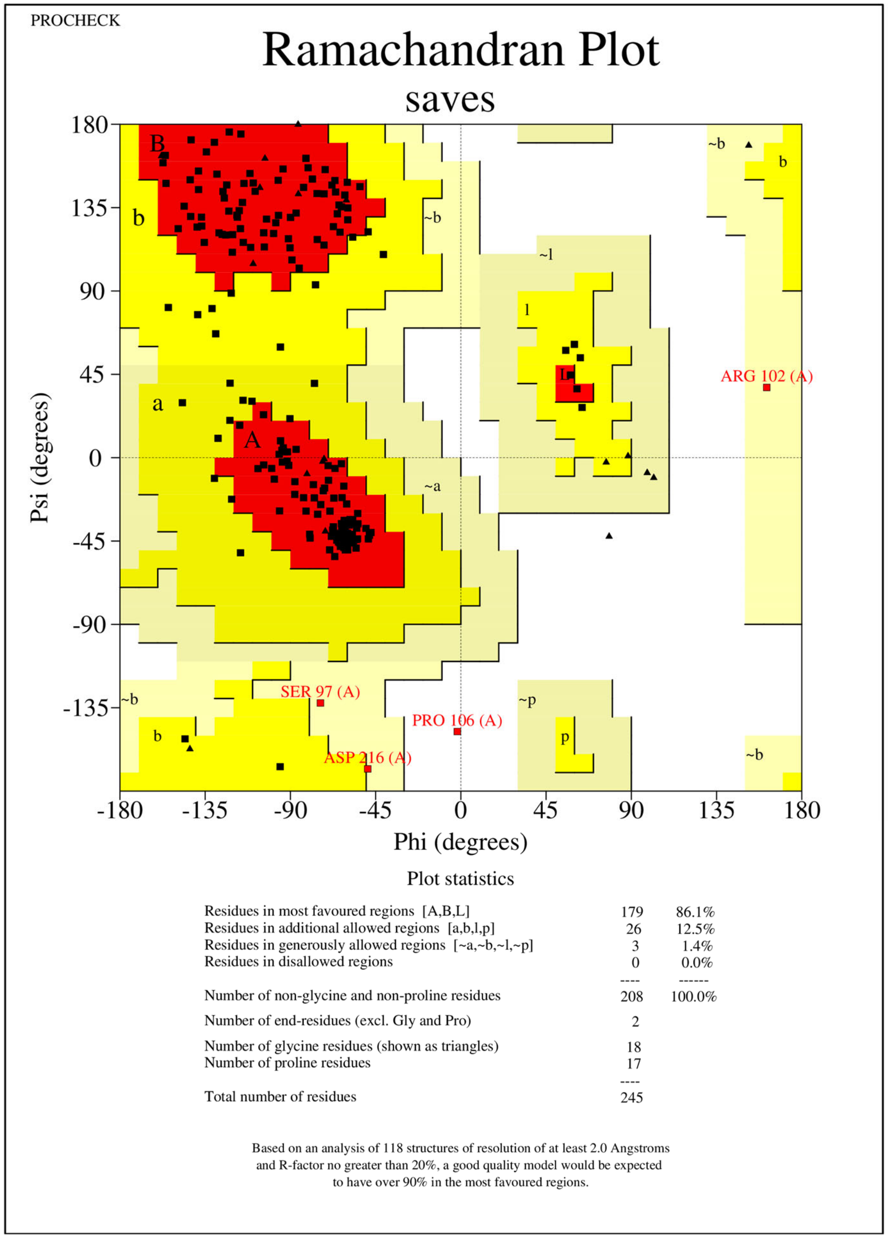
PROCHECK
Ramachandran Plot
saves
Psi (degrees)
Phi (degrees)
ARG 102 (A)
SER 97 (A)
PRO 106 (A)
ASP 216 (A)
Plot statistics
Residues in most favoured regions [A,B,L] 179 86.1%
Residues in additional allowed regions [a,b,l,p] 26 12.5%
Residues in generously allowed regions [~a,~b,~l,~p] 3 1.4%
Residues in disallowed regions 0 0.0%
Number of non-glycine and non-proline residues 208 100.0%
Number of end-residues (excl. Gly and Pro) 2
Number of glycine residues (shown as triangles) 18
Number of proline residues 17
Total number of residues 245
Based on an analysis of 118 structures of resolution of at least 2.0 Angstroms and R-factor no greater than 20%, a good quality model would be expected to have over 90% in the most favoured regions.

(A)

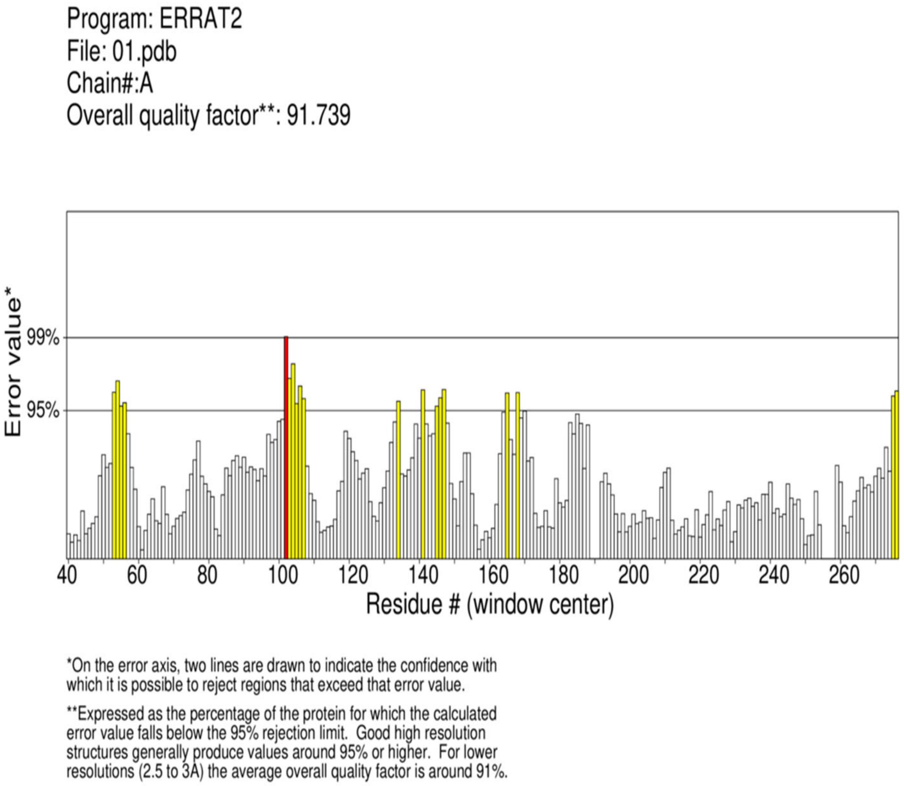
Program: ERRAT2
File: 01.pdb
Chain#:A
Overall quality factor**: 91.739
Error value*
99%
95%
Residue # (window center)
*On the error axis, two lines are drawn to indicate the confidence with which it is possible to reject regions that exceed that error value.
**Expressed as the percentage of the protein for which the calculated error value falls below the 95% rejection limit. Good high resolution structures generally produce values around 95% or higher. For lower resolutions (2.5 to 3A) the average overall quality factor is around 91%.

(B)

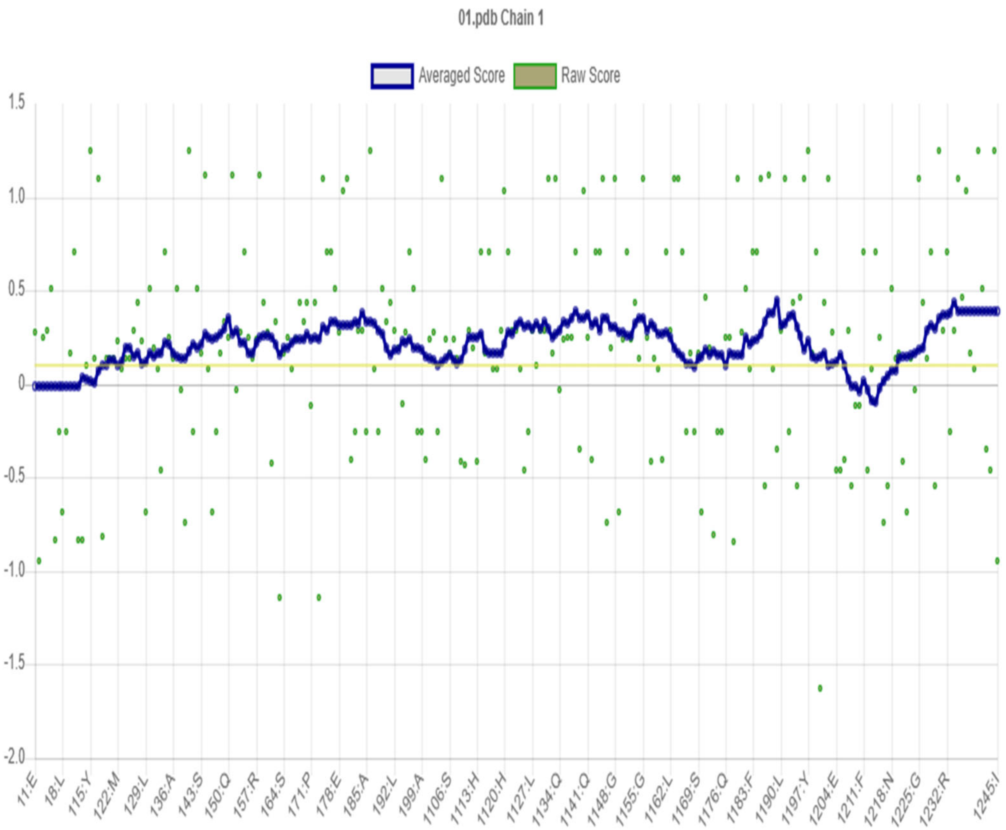

(**C**)

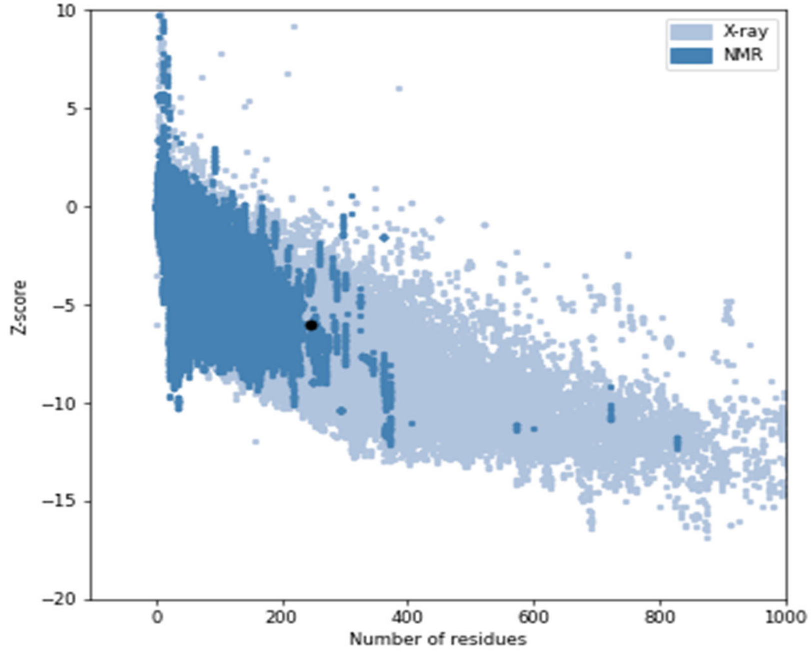

(**D**)

Figure S4. Model validation results: **(A)** Ramachandran plot analysis, **(B)** ERRAT evaluation, **(C)** VERIFY-3D assessment, and **(D)** ProSA analysis.

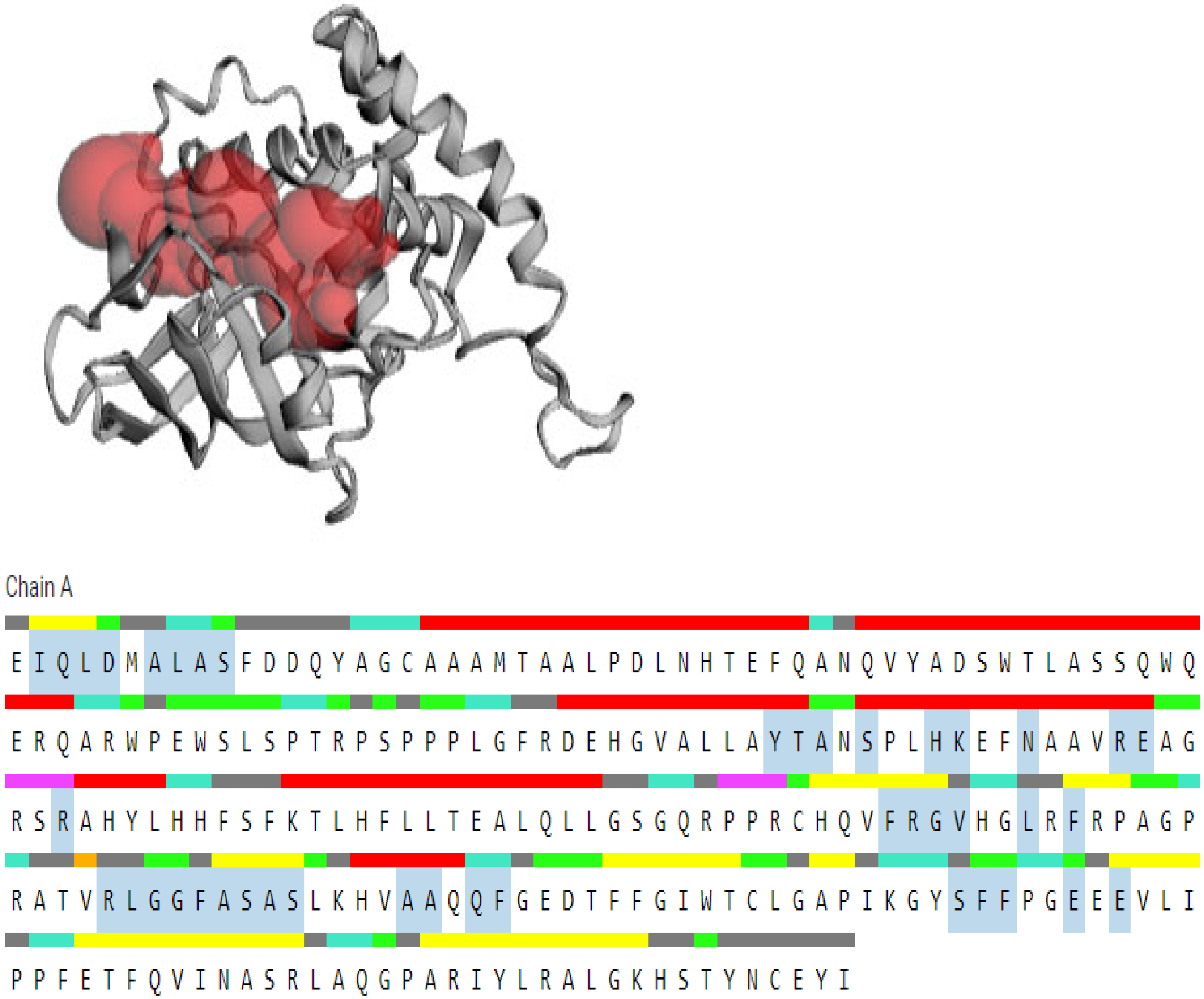

Figure S5. Active site of the predicted protein. (A) The pocket of the protein's active site. (B) The marked amino acid residues in the active site of protein

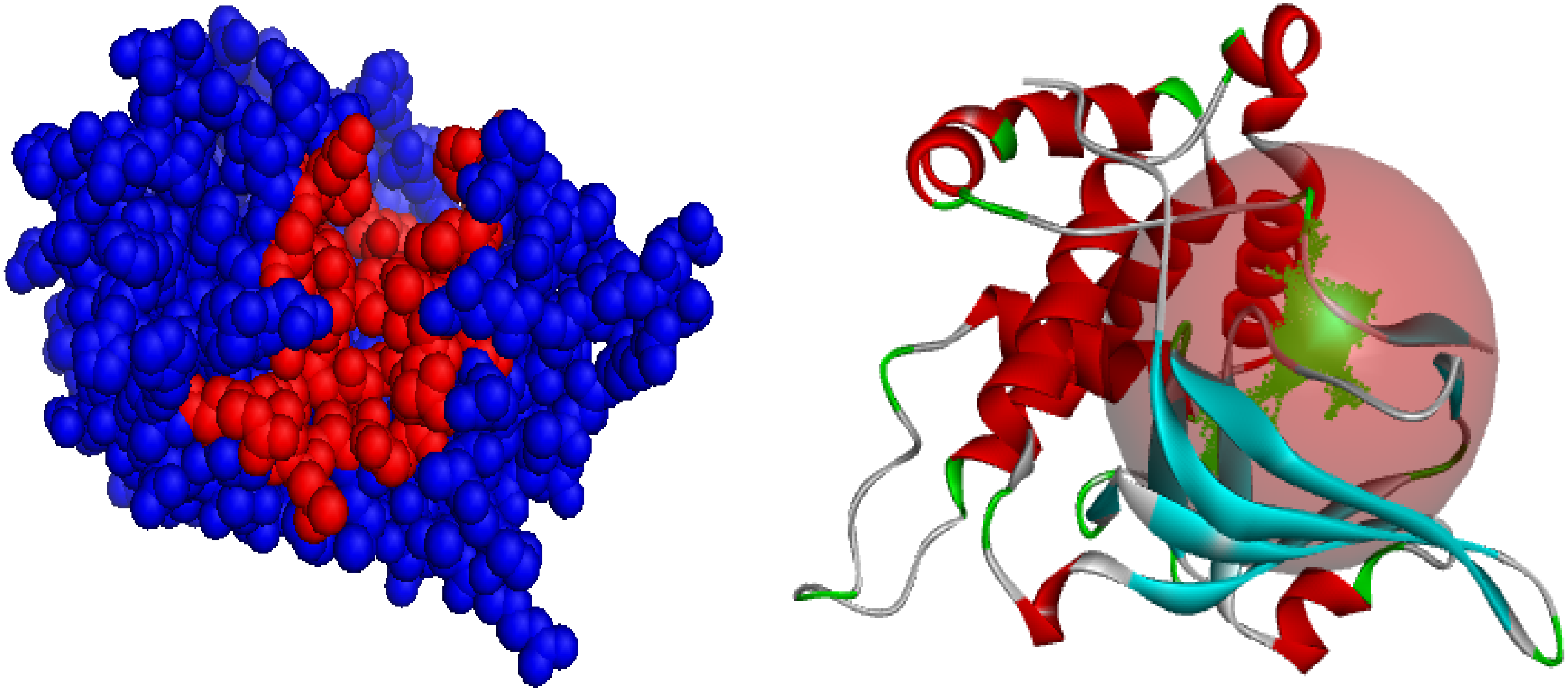

Figure S6. Visualization of the predicted protein's active site using PyMOL and Discovery Studio.

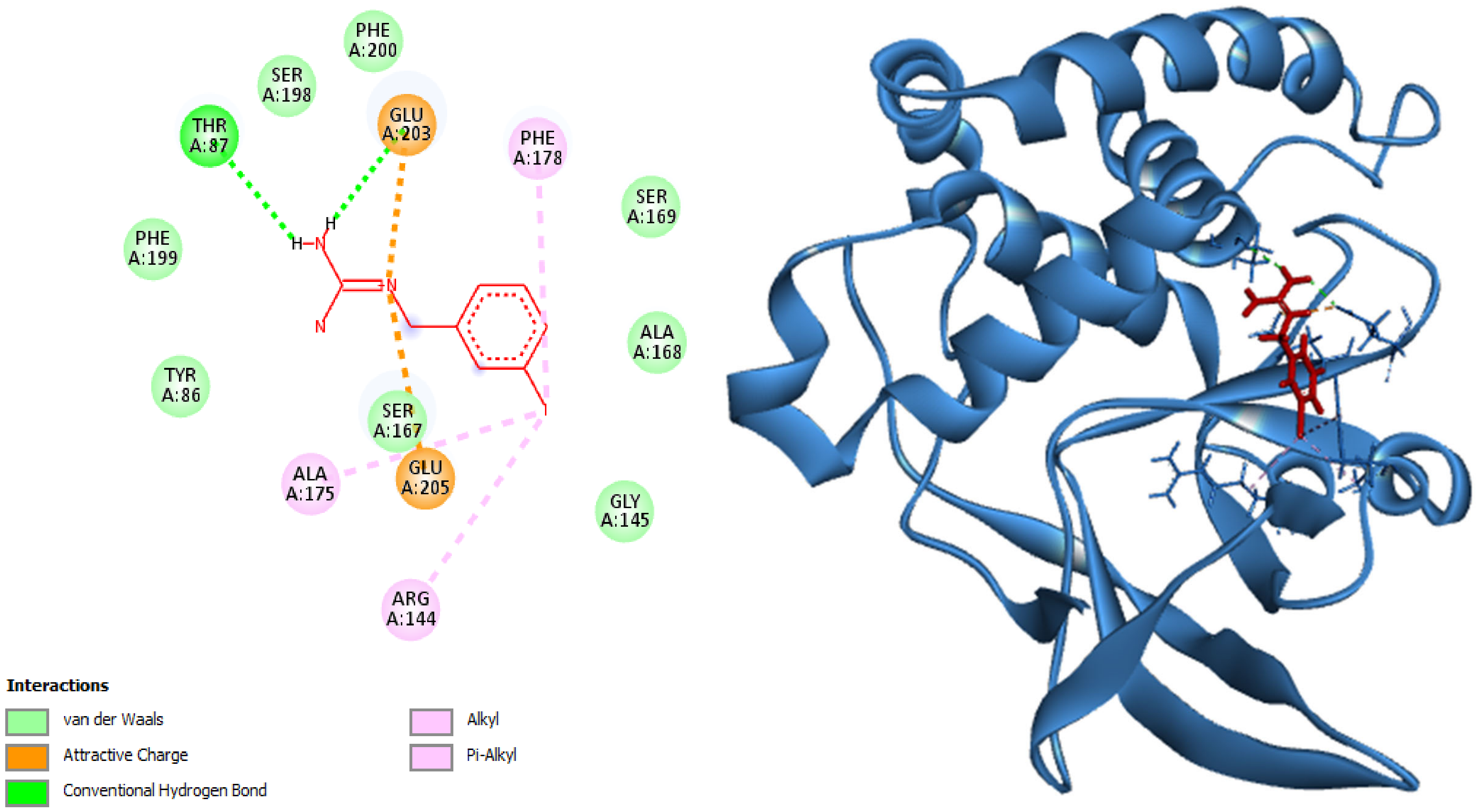

ART1-meta-iodobenzylguanidine

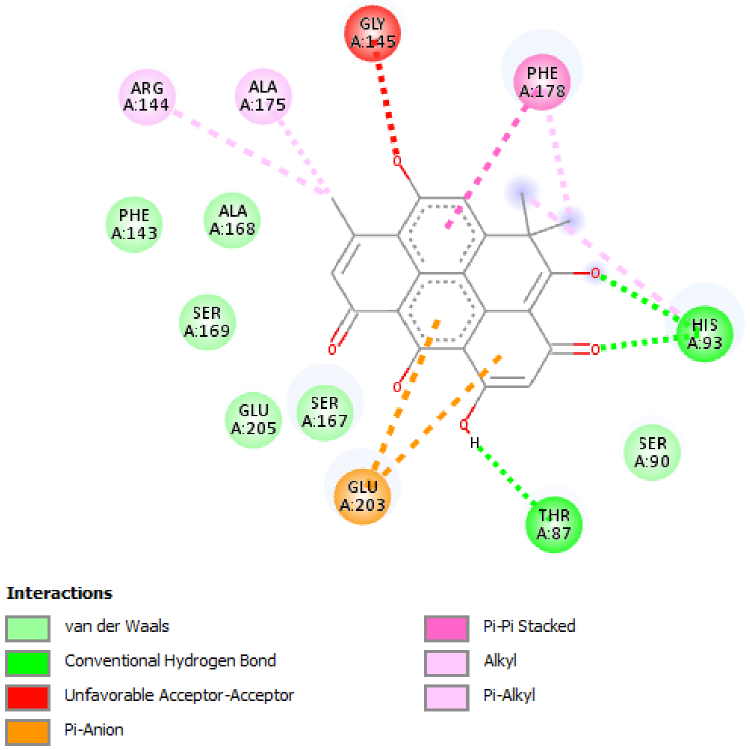

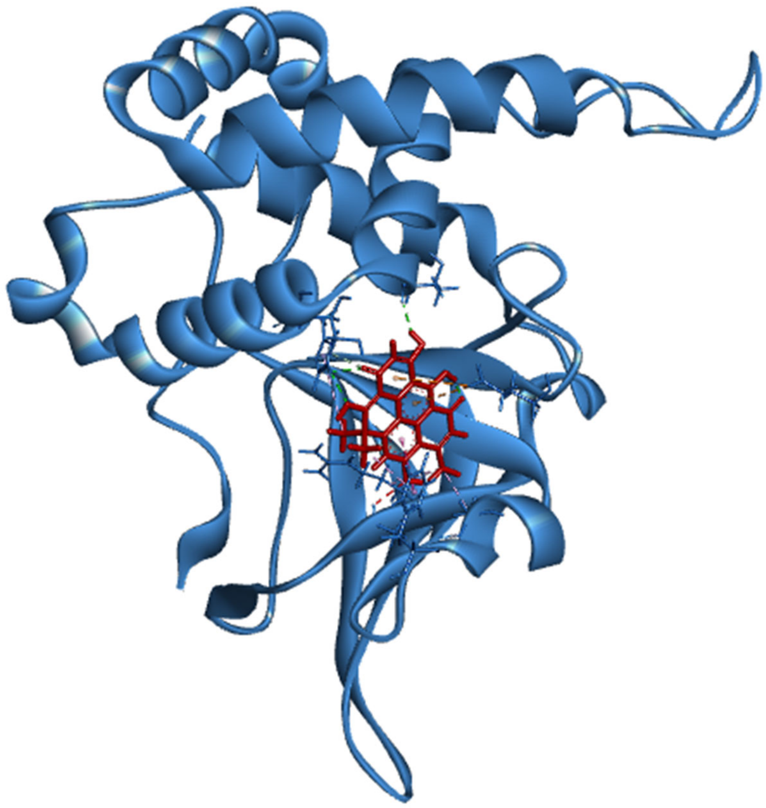

ART1-Resistomycin

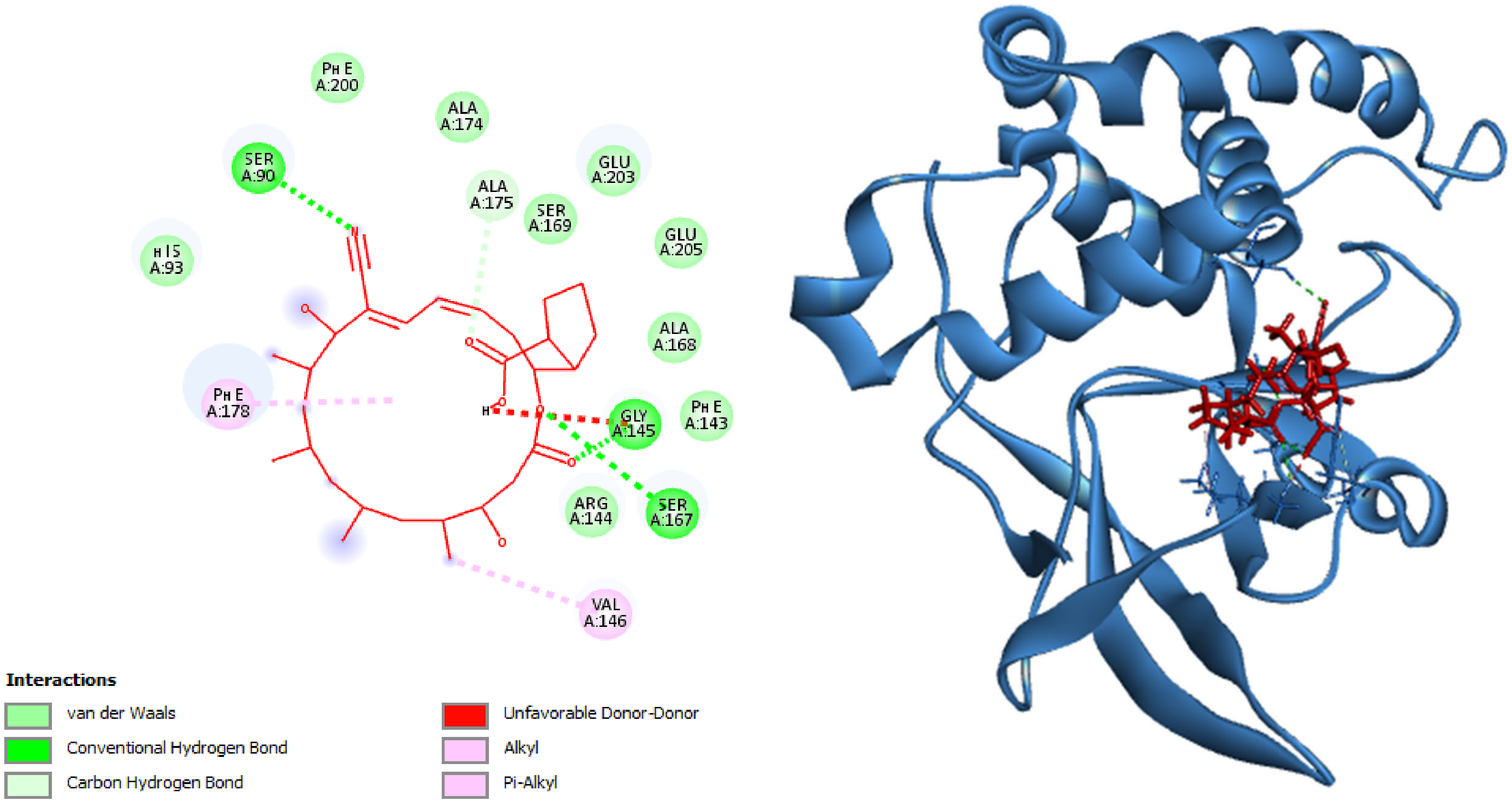

ART1-Borrelidin

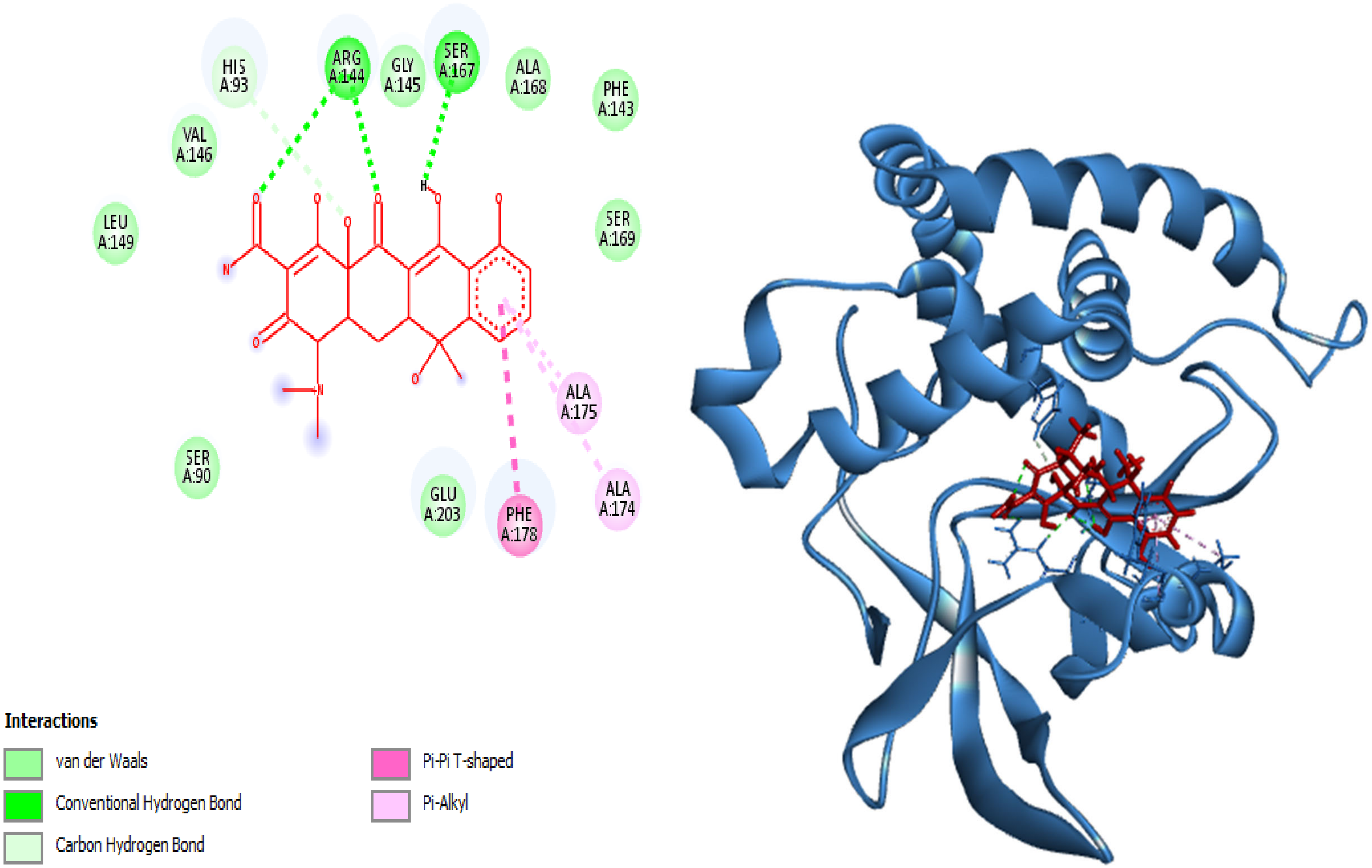

ART1-Tetracycline

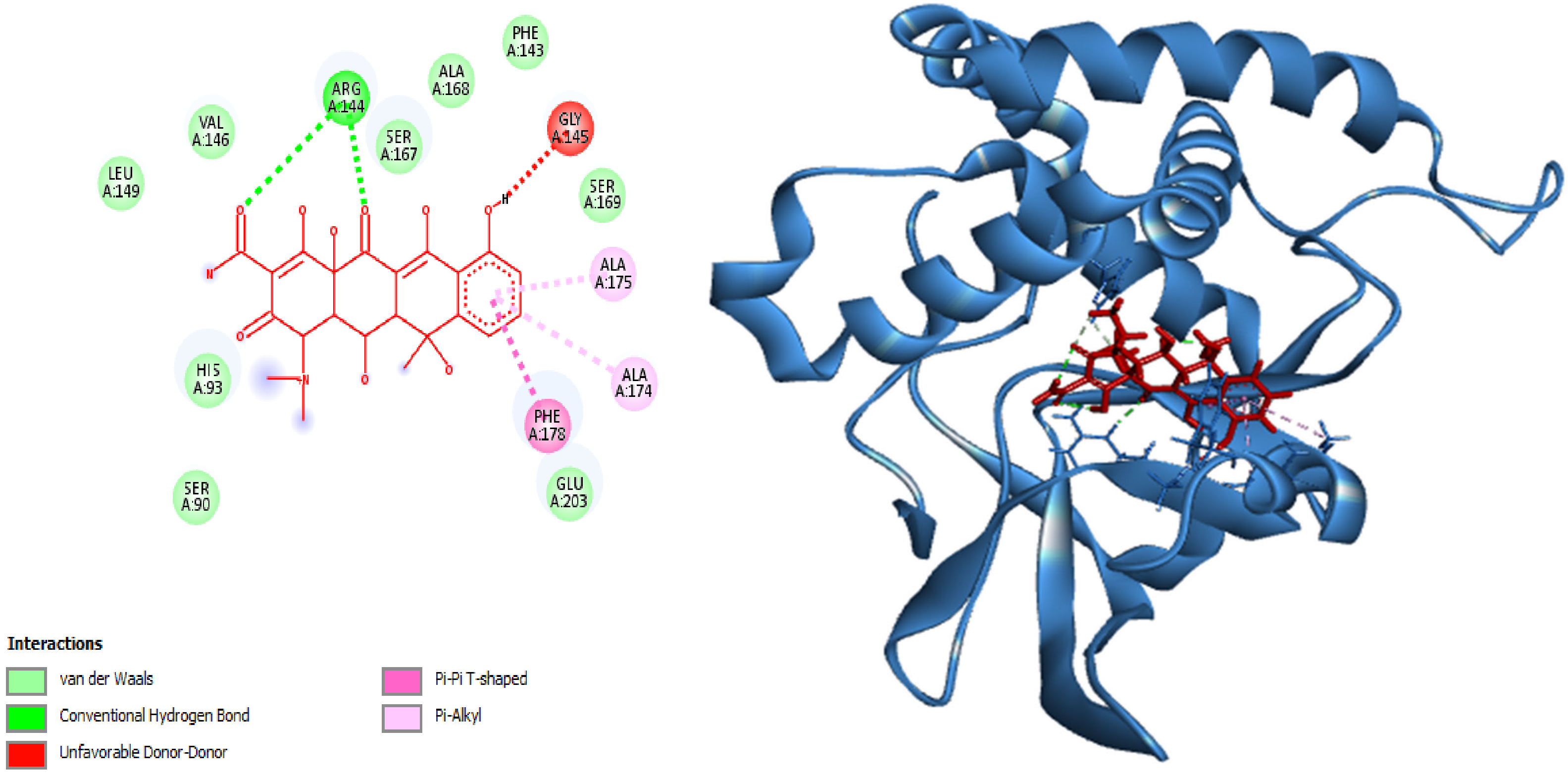

ART1-Oxytetracycline

Figure S7. Two- and three-dimensional visualizations of the interactions between ART1 (in blue) and the top-ranked ligands (in red), emphasizing key binding features.
